# Organocatalyzed Atom Transfer Radical Polymerization (O‐ATRP) Using a Super‐Reducing Photoredox Catalyst

**DOI:** 10.1002/anie.202517641

**Published:** 2025-10-08

**Authors:** Yucheng Zhao, Brandon S. Portela, Alexander R. Green, Anna M. Wolff, Xin Liu, Katherine O. Puffer, Arindam Sau, Niels H. Damrauer, Robert S. Paton, Garret M. Miyake

**Affiliations:** ^1^ Department of Chemistry Colorado State University Fort Collins CO 80523 USA; ^2^ Department of Chemistry University of Colorado Boulder Boulder CO 80309 USA; ^3^ Renewable and Sustainable Energy Institute (RASEI) University of Colorado Boulder Boulder CO 80309 USA

**Keywords:** O‐ATRP, Photocatalyst, Photoredox, Super‐reducing, SuPRCat

## Abstract

The development of highly reducing photoredox catalysts (PCs) has brought forth new approaches to activating strong chemical bonds for small molecule and polymer synthesis. Organocatalyzed atom transfer radical polymerization (O‐ATRP) is a polymerization methodology that uses organic PCs for the synthesis of well‐defined polymers proceeding through a reversible‐deactivation mechanism. However, the reaction scope of O‐ATRP is confined to initiators and dormant polymer states that can be reduced by a PC. Herein, we report an O‐ATRP system using a super‐reducing PC to expand the capability of O‐ATRP to monomers (such as styrene (St) and vinylcarbazole (VCz)) and initiators (including aromatic halides and pseudo‐halides) that are challenging with most O‐ATRP systems. This system provides control over polymerization, air tolerance, and temporal regulation and enables the synthesis of polymer brushes via organocatalyzed grafting‐from reactions on linear chains. This strategy builds upon the transformative potential of super‐reducing PCs toward advancing the field of reversible‐deactivation radical polymerization.

## Graphical abstract

A super‐reducing organic photocatalyst expands the scope of O‐ATRP to challenging initiators and monomers. The system enables controlled polymerizations with air tolerance, temporal regulation, and grafting‐from strategies, highlighting the transformative potential of super‐reducing PCs in reversible‐deactivation radical polymerization.

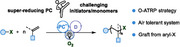

O‐ATRP is a polymerization methodology utilizing organic photoredox catalysts.^[^ ^1^, ^2^, ^3^ ^]^ As a variant to traditional metal‐mediated ATRP,^[^ ^4^ ^]^ O‐ATRP allows for the synthesis of well‐defined polymers with low dispersity and controlled topological structures, while eliminating concerns over transition‐metal contamination.^[^ ^5^, ^6^ ^]^ O‐ATRP leverages organic PCs to activate alkyl halide initiators and dormant polymer halides via photoinduced single electron transfer (SET)‐initiated mesolytic cleavage, offering the additional benefits of temporal and spatial control.^[^ ^1^ ^]^ Over the last decade, numerous PCs for the O‐ATRP of methyl methacrylate (MMA) have been developed (Figure 1a).^[^ ^7^, ^8^, ^9^, ^10^, ^11^ ^]^ The scope of initiators and monomers in O‐ATRP are limited by reduction potentials (*E*_red_), where *E*_red_ for initiators and dormant species (*E*_1/2_(RBr/RBr^•−^) or *E*_1/2_(P*_n_*‐Br/P*_n_*‐Br^•−^)) are typically around −0.8 V versus SCE.^[^ ^1^ ^]^ Initiators and dormant polymer halides with more negative reduction potentials, such as polystyrene bromide (PS‐Br), which has a reduction potential below −1.5 V versus SCE,^[^ ^9^ ^]^ remain challenging to activate. Expanding the range of suitable monomers and initiators will further enable the exploration of polymer properties and new applications of O‐ATRP.^[^ ^12^, ^13^, ^14^, ^15^ ^]^

**Figure 1:**
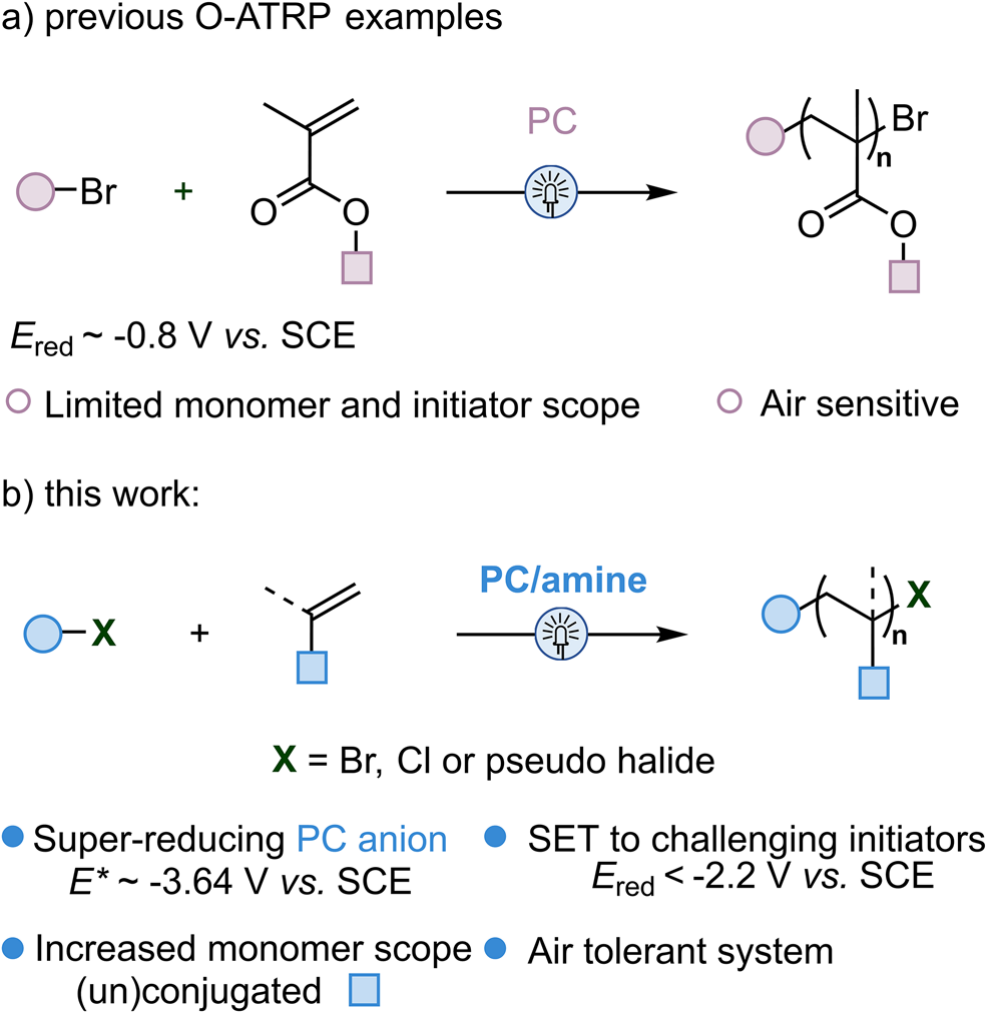
a) O‐ATRP with conventional reducing catalysts enables polymerization of common initiators and monomers (methacrylates). b) This work employs a super‐reducing, air‐tolerant catalyst system to access challenging initiators and monomers.

To address this challenge, PCs are needed that have increased reduction power while maintaining their ability to mediate both the activation and deactivation steps. However, this extension is challenging as the reducing power of photoredox catalysis using blue light is limited by the stored energy (*E*_00_) of approximately 2.8 eV.^[^ ^16^ ^]^ To overcome the energetic limitations, anionic PCs have emerged as promising candidates, benefiting from their increased electron density for impacting the reduction potentials.^[^ ^17^ ^]^ Recently, anionic radical PCs (PC^·−^) that can harness the energy of two photons to drive high‐energy chemical transformations have been developed.^[^ ^16^, ^18^, ^19^, ^20^, ^21^, ^22^ ^]^ This concept has been applied to O‐ATRP using a perylenetetracarboxylic diimide (*E*_1/2_(PC/PC^·−^*) = −1.87 V versus SCE) as the PC, ultimately expanding the initiator scope to include aromatic bromides and enabling controlled polymerization of MMA.^[^ ^23^ ^]^ Inspired by these advancements, and aiming to further enhance reduction power and oxygen tolerance, we sought to utilize an anionic PC, generated via photoinduced sequential two‐electron reduction followed by protonation (2e^−^/1H^+^),^[^ ^24^, ^25^, ^26^, ^27^ ^]^ for O‐ATRP. In our previous work, benzo[*ghi*]perylene monoamide (**BPI**) undergoes ring‐opening, followed by 2e^−^/1H^+^‐reduction to generate **BPI‐RO‐H^2^** ^−^, which has been demonstrated in arene reduction and C─F bond activation,^[^ ^25^, ^26^, ^27^, ^28^ ^]^ possessing a potent excited state reduction potential of −3.64 V versus SCE.^[^ ^26^ ^]^ As such, we hypothesized this super‐reducing PC would allow for use of more inert initiators and polymerization of an expanded scope of monomers by O‐ATRP (Figure 1b).

Our approach to developing a new catalytic system for O‐ATRP aimed not only to overcome the current limitations in activating challenging initiators and monomers but also to ensure well‐controlled polymerization through efficient deactivation. We proposed that the super‐reducing species could be formed with amines serving as both electron donors and as proton donors through their radical cations (D^•^⁺).^[^ ^24^, ^26^ ^]^ Irradiating a mixture of **BPI** and 50 equiv of amine (e.g., tribenzylamine, TBnA) in dimethylacetamide (DMAc) with 450 nm LED light for 3 h generates the super‐reducing PC (Figures 2a,b and 4).^[^ ^26^ ^]^ This species could then activate challenging initiators or P*_n_*–Br through SET from its excited state. Meanwhile, the amine could help regenerate the PC, and the resultant D^•^⁺ would assist in converting propagating chains back into dormant species, thereby maintaining polymerization control (Figure 4). Leveraging the air tolerance imparted by amines in ATRP,^[^ ^29^, ^30^ ^]^ this strategy would expand the monomer and initiator scopes in O‐ATRP while exhibiting air tolerance.

**Figure 2:**
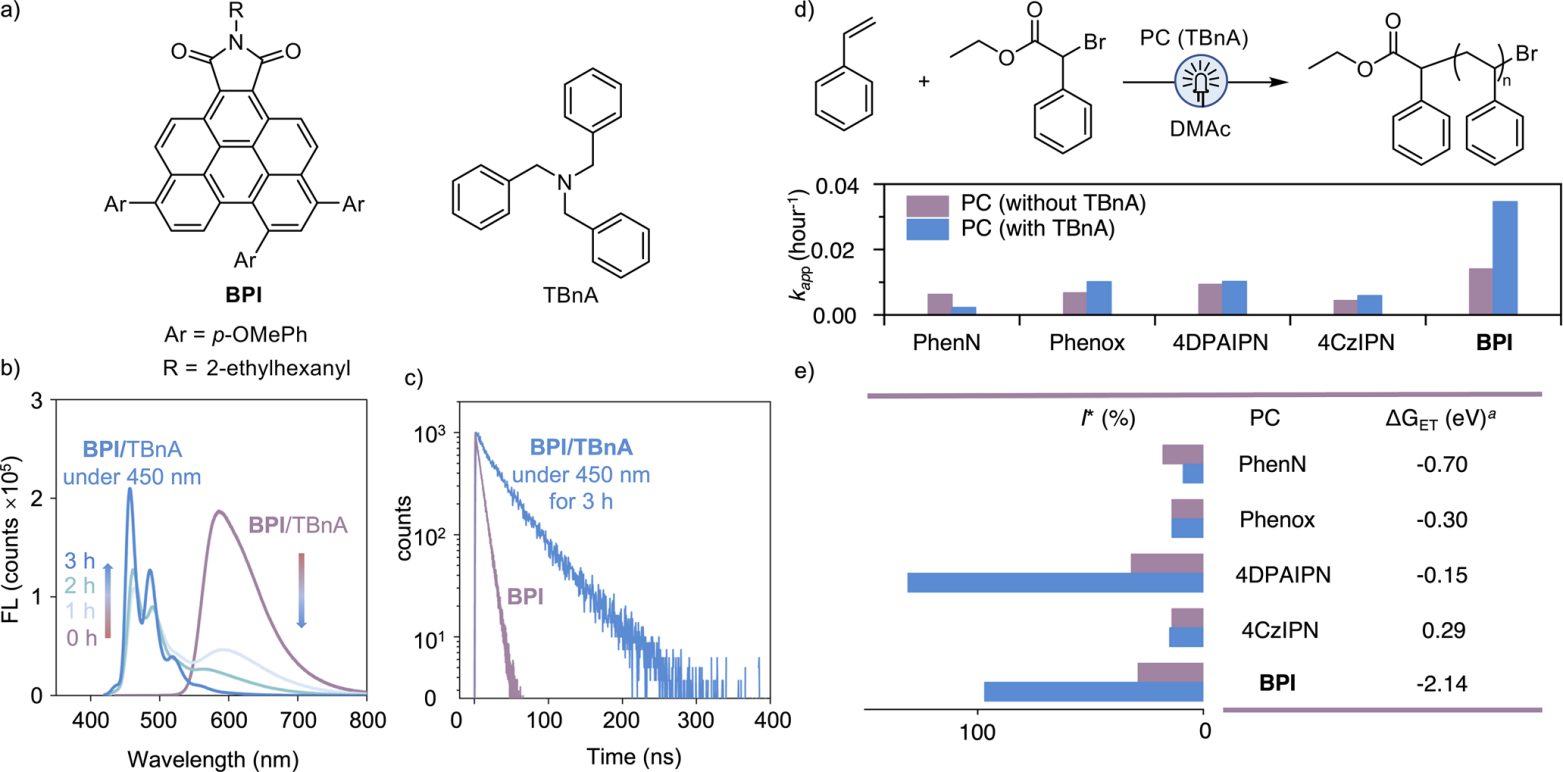
The O‐ATRP of styrene (St) using different types of organic photocatalysts. a) Structures of **BPI** and electron donor. b) Fluorescence spectra of the **BPI**/tribenzylamine (TBnA) in DMAc under 450 nm light irradiation from 0 to 3 h. c) The transient decay curves of **BPI**/TBnA treated with 450 nm light for 3 h and BPI in DMAc. d) O‐ATRP of St. Conditions: 4.00 mmol St, [St]/[EBP]/[PC] = 200/1/0.02 in DMAc, [EBP]:[TBnA] = 1:5 when TBnA was used, 450 nm LED light, 25 °C, 48 h. Commercially available PCs: PhenN, 5,10‐di(naphthalen‐2‐yl)‐5,10‐dihydrophenazine, Phenox, 3,7‐di([1,1′‐biphenyl]‐4‐yl)‐10‐(naphthalen‐1‐yl)‐10*H*‐phenoxazine, 4DPAIPN, 2,4,5,6‐tetrakis(diphenylamino)isophthalonitrile, 4CzIPN, 1,2,3,5‐tetrakis(carbazol‐9‐yl)‐4,6‐dicyanobenzene. Apparent rate constants (*k*_app_) were estimated based on the slope of the kinetic studies for PCs in the presence/absence of electron donors used in the O‐ATRP. e) Initiator efficiency (I*), free energy for the electron transfer (ET) reaction (Δ*G*_ET_), and fluorescence lifetimes of the PCs. ^a)^Δ*G*_ET_ was estimated by Rehm–Weller equation and the *E*_red_ (PS‐Br) ∼ −1.5 V versus SCE was used.^[^ ^9^ ^]^

The O‐ATRP of St irradiated with 450 nm light was performed using **BPI** as the catalyst and ethyl 2‐bromo‐2‐phenylacetate (EBP) as the initiator. Various amines (e.g., triethylamine (TEA) and *N*,*N*‐diisopropylethylamine) were examined as electron donors (D) to investigate their impact on the polymerization (Table S1). Notably, when 5 equiv of TBnA (*E*_ox_ = 1.03 V versus SCE)^[^ ^31^ ^]^ relative to the initiator was used, the polymerization achieved a conversion of 82% with a high initiator efficiency (I* = 96%). Unlike TEA, TBnA generates an α‐amino radical that is essentially unreactive toward olefins, thereby minimizing the formation of new polymer chains (Figure 4).^[^ ^32^ ^]^ Under otherwise identical conditions, the performance of various PCs, including 5,10‐di(naphthalen‐2‐yl)‐5,10‐dihydrophenazine (PhenN), 3,7‐di([1,1′‐biphenyl]‐4‐yl)‐10‐(naphthalen‐1‐yl)‐10*H*‐phenoxazine (Phenox), 2,4,5,6‐tetrakis(diphenylamino)isophthalonitrile (4DPAIPN), and 1,2,3,5‐tetrakis(carbazol‐9‐yl)‐4,6‐dicyanobenzene (4CzIPN), in the O‐ATRP of St was assessed. The apparent rate constants (*k*_app_) (Figures 2d and S1–S8) for PhenN, Phenox, 4DPAIPN, and 4CzIPN remained low (<1.0 × 10^−^^2^ h^−1^), whereas **BPI**/TBnA exhibited a higher *k*_app_ of 3.6 × 10^−^^2^ h^−1^ (Figure 3a and Table S3). The greater free energy of electron transfer (Δ*G*_ET_) between BPI‐derived PC and PS‐Br (−2.14 eV versus SCE, Table S3) led to a faster propagation rate and higher I* (Figure 2e). By comparison, the other PCs have Δ*G*_ET_ ranging from −0.70 to 0.29 eV versus SCE.

**Figure 3:**
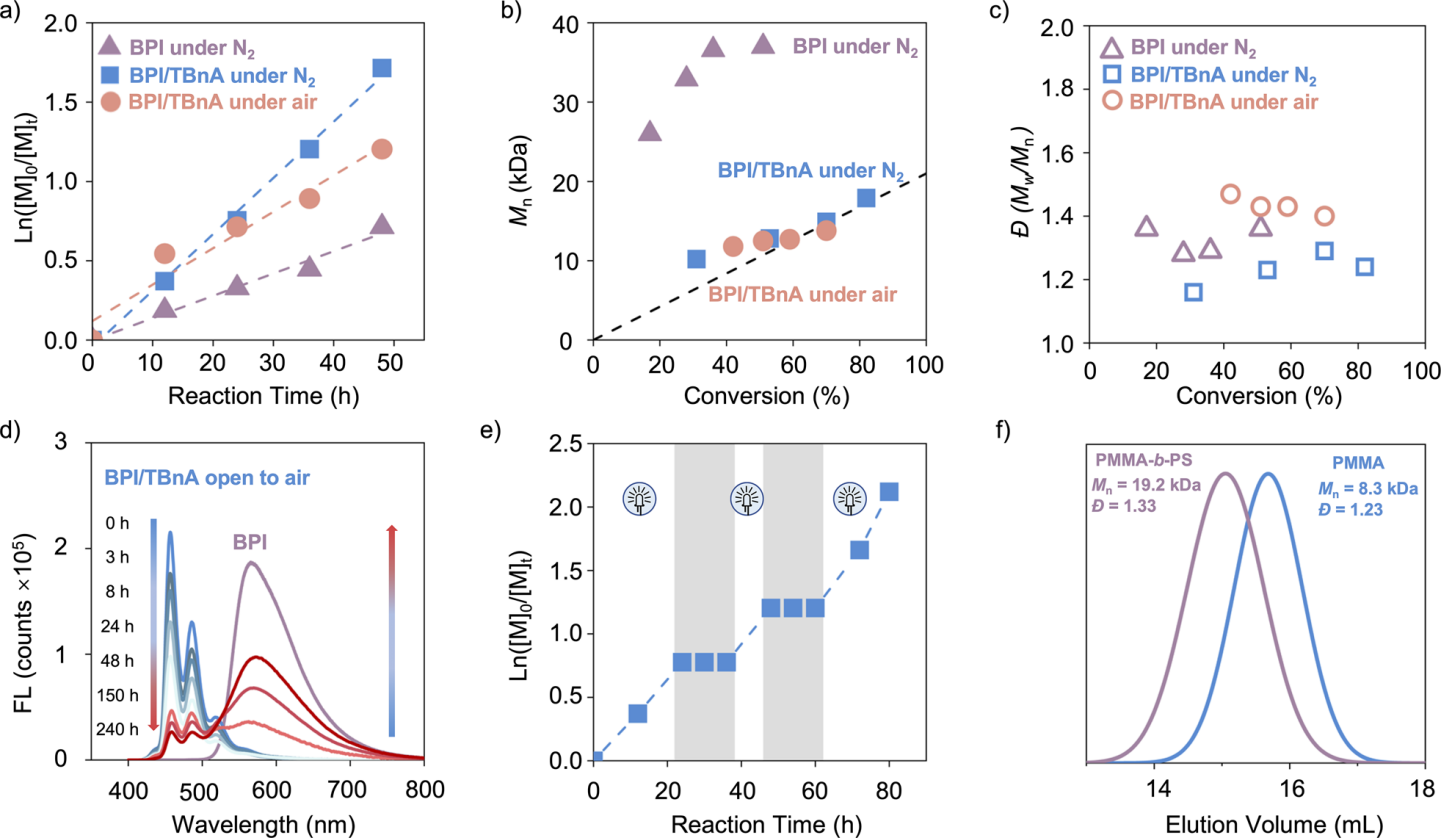
O‐ATRP of St using **BPI** as PC. a) Kinetic studies under N_2_ and air atmosphere, respectively. b) *M*_n_ as a function of conversion under N_2_ and air atmosphere, respectively. c) *Đ* as a function of conversion under N_2_ and air atmosphere, respectively. d) The fluorescence spectra of **BPI**/TBnA in DMAC open to air for 0–240 h. e) Temporal control of O‐ATRP. f) Chain extension of a PMMA macroinitiator with St.

Without an initiator, the reaction with St reached 50% conversion producing a polymer with low number average molecular weight (*M*_n_ = 6.1 kDa) and high dispersity (*Đ* = 2.01, Figure S10 and Table 1), suggesting that the BPI‐derived PC can directly reduce St (*E*_red_ = −2.57 V versus SCE).^[^ ^33^ ^]^ When a low catalyst loading (100 ppm) was used in the presence of the initiator EBP, the polymerization proceeded with good control, yielding a low *Đ* (1.24) and high I* (96%, entry 2, Table 1). The experimentally measured *M*_n_ values aligned closely with the theoretical molecular weight *M*_n,theo_ (Figure 3b), indicating a well‐controlled radical polymerization process. Even at just 5 ppm of catalyst, *M*_n_ increased linearly with conversion. However, increasing the catalyst loading to 1000 ppm did not significantly accelerate the polymerization and resulted in a decrease in control over the polymerization (Figure S11), likely due to the direct reduction of monomer or increased chain termination. When using 100 ppm of **BPI**, the polymerization was controlled when varying the [St]:[EBP] ratios to 100:1 and 400:1 (Figures S12, S13 and Table 1). Increasing the polymerization temperature to 50 °C enhanced efficiency (Figure S14 and Table 1), although it compromised control over the polymerization.

**Table 1:** Results for the O‐ATRP of different monomers.^**a)**^

Entry	M	[M]:[EBP]:[**BPI**]	Conv. (%)	*M*_n,theo_ (kDa)	*M*_n_ (kDa)	*Đ*	I* (%)
1	St	200:0:0.02	50	–	6.1	2.01	–
2	St	200:1:0.02	82	17.2	17.9	1.24	96
3	St	200:1:0.2	89	18.7	19.0	1.25	98
4	St	200:1:0.001	27	5.9	45.8	1.23	13
5	St	100:1:0.01	79	8.4	13.0	1.35	65
6	St^b)^	400:1:0.04	76	31.9	32.4	1.21	98
7	St^c)^	400:1:0.04	89	40.8	55.3	1.29	74
8	4FSt	200:1:0.02	85	21.0	26.8	1.36	78
9	4CSt	200:1:0.02	72	20.1	26.9	1.21	75
10	MMA^d)^	200:1:0.02	93	18.8	20.3	1.33	93
11	MA^e)^	200:1:0.02	86	15.0	15.5	2.22	97
12	VCz^f)^	200:1:0.02	70	27.3	88.9	1.49	31

^a)^ Conditions: 4.00 mmol monomer in 500 µL DMAc, [EBP]/[TBnA] = 1/5, 450 nm LED light, 25 °C, ambient pressure, 48 h. Conversions were based on ^1^H NMR analysis of the remaining monomer after reactions. *M*_n,theo_ were calculated based on monomer conversions. *M*_n_ and *Đ* were measured by SEC‐MALS (tetrahydrofuran). I* = (*M*_n,theo_/*M*_n_) × 100%. 4FSt, 4‐fluorostyrene, 4CSt, 4‐chlorostyrene, MMA, methyl methacrylate, MA, methyl acrylate, VCz, vinylcarbazole.

^b)^ Reaction time = 60 h.

^c)^ At 50 °C, 48 h.

^d)^ Reaction time = 6 h.

^e)^ Reaction time = 2 h.

^f)^ Reaction time = 10 h.

Due to the super‐reducing nature of the catalyst we hypothesized that this system could efficiently activate challenging initiators (e.g., benzyl chloride, BnC, *E*_red_ = −2.21 V versus SCE,^[^ ^34^ ^]^ and (1‐bromoethyl)benzene, PEBr, *E*_red_ = −1.60 V versus SCE^[^ ^35^ ^]^), while achieving a well‐controlled polymerization. Polymerization of St using common alkyl halides (Figures S23, S24 and Table 2, entries 1–4, 6, and 7) were well‐controlled producing polymers with low *Đ* (1.24–1.31) and achieving good to high I* (64–96%). Even for more difficult‐to‐reduce initiators, such as an aromatic bromide (1‐bromo‐4‐(trifluoromethyl)benzene, BFTB, *E*_red_ ∼−2.4 V versus SCE entry 5, Table 2), although the conversion was lower (60%), the I* remains high (98%). The activation of BTFB by **BPI**/TBnA was also supported by the Stern–Volmer quenching and the ─CF_3_ signals remained after purification of polymer in the ^19^F NMR spectrum (Figures S25 and S26). In contrast, the polymerization using aromatic chloride (*E*_red_ ∼−2.8 V versus SCE),^[^ ^19^ ^]^ which is more difficult to reduce than St (*E*_red_ = −2.57 V versus SCE), did not show a linearly increasing *M*_n_ with monomer conversion (Figure S27). This result suggests that initiation can also occur from the monomer, leading to poor control over polymerization. Pseudo‐halides applied in O‐ATRP have been limited due to the significantly lower activity of the transfer group.^[^ ^36^ ^]^ Using this catalyst system, a pseudo halide (alpha‐methylbenzyl isothiocyanate, BESCN) showed moderate initiation in the O‐ATRP of St with 50% conversion and 51% I*. The *M*_n_ also increased linearly with increasing conversion, indicating some control (Figure S28). Styrene derivatives, MMA, and methyl acrylate (MA) were polymerized with I* ranging 75%–97% (Figures S15–S18). Even unconjugated monomers such as vinylcarbazole could be polymerized, albeit with low I* values (31%) (Figures S20 and S36).

**Table 2:** O‐ATRP of St using different initiators.^a)^

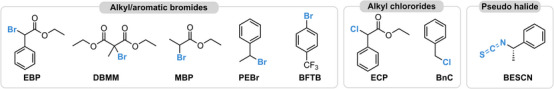						
Entry	Initiator	Conv. (%)	*M*_n,theo_ (kDa)	*M*_n_ (kDa)	*Đ*	I* (%)
1	EBP	82	17.2	17.9	1.24	96
2	DBMM	76	15.2	17.6	1.28	86
3	MBP	85	17.9	19.6	1.27	91
4	PEBr	80	16.9	22.7	1.29	74
5	BTFB	60	12.7	12.9	1.34	98
6	ECP	70	14.8	19.1	1.25	77
7	BnC	89	18.8	29.3	1.31	64
8	BESCN	55	11.7	22.9	1.36	51

^a)^ [St]/[initiator]/[**BPI**]/[TBnA] = 200/1/0.02/5 in DMAc, 450 nm LED light, 25 °C, ambient pressure, 48 h. Conversions were based on ^1^H NMR analysis of the remaining St after reactions. *M*_n,theo_ were calculated based on St conversions. *M*_n_ and *Đ* were measured by SEC‐MALS.

Polymerization kinetics in the absence of TBnA were monitored (Figure 3). Compared to the polymerization in the presence of TBnA, the *k*_app_ values were lower, and the molecular weights deviated from the *M*_n,theo_, underscoring the critical role of the donor in the polymerization. Under air, the conversion for O‐ATRP of St reached 70%, and the process remained controlled (I* = 108%, *Đ* = 1.40), although the polymerization rate was slower than that observed under nitrogen (Figure 3a). **BPI**/TBnA showed good air‐tolerant behavior (Figures 3d and S29). The formation of amides on TBnA suggests the role of amine in enabling air tolerance in O‐ATRP (Figures 4 and S30).^[^ ^29^, ^30^ ^]^ All conditions showed pseudo‐first‐order kinetics. Furthermore, this O‐ATRP system demonstrated good temporal control during two ON/OFF cycles, with polymerization reactivating even after a long OFF period (12 h), indicating efficient reactivation of the active species (Figure 3e). Structural analysis of resulting polymers by ^1^H NMR spectroscopy and matrix‐assisted laser desorption ionization time of flight (MALDI‐TOF) mass spectrometry confirmed the presence of chain‐end functionality (Figures S31–S33). Additionally, chain extension was successfully performed maintaining good chain‐end fidelity of the resulting polymer (Figures 3f and S34).

**Figure 4:**
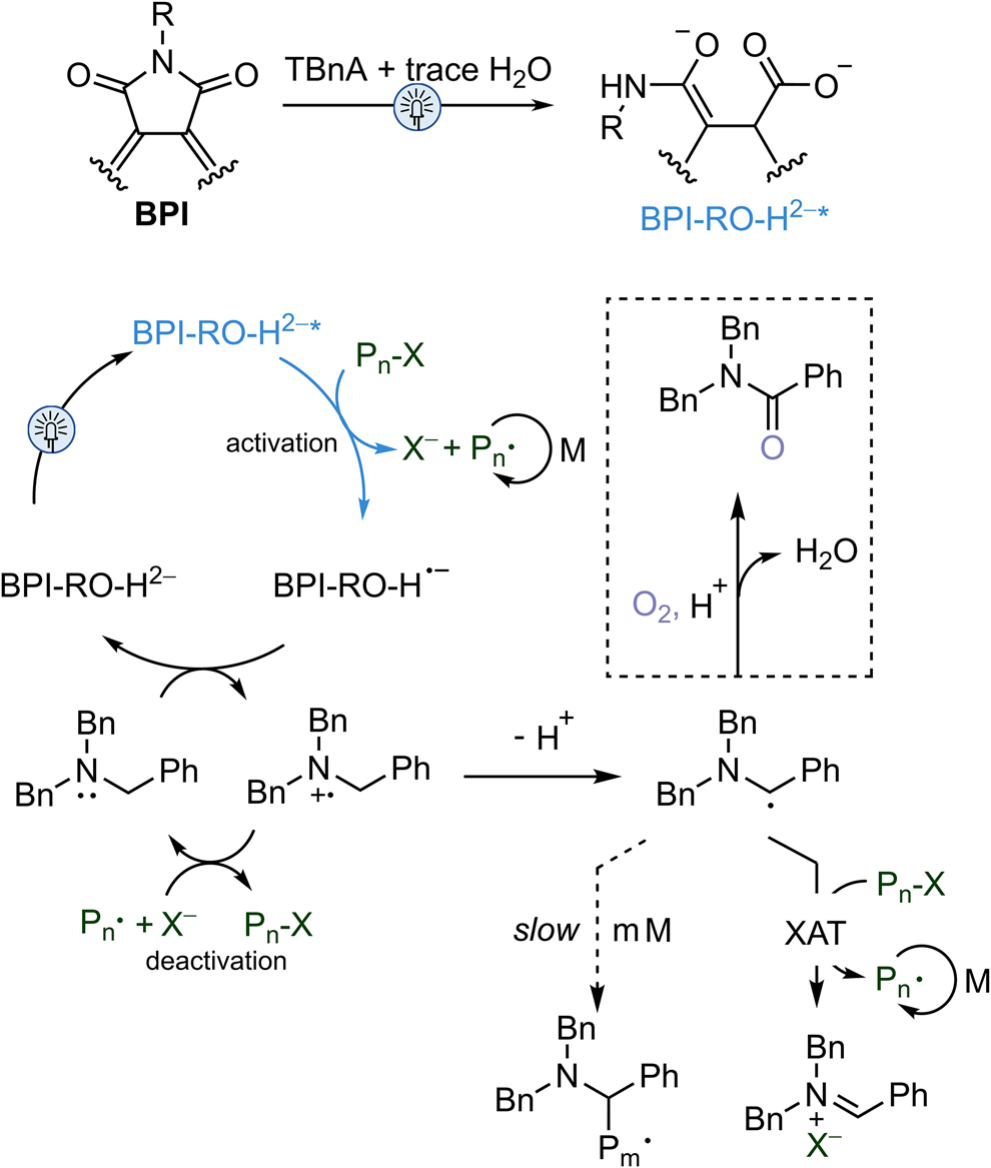
Proposed mechanisms for the formation of super‐reducing catalyst,^[^ ^26^, ^27^ ^]^ and O‐ATRP system mediated by BPI and TBnA.^[^ ^29^, ^30^, ^32^ ^]^

Lastly, leveraging the well‐controlled polymerization of St and the expanded initiator scope, we envisioned that this O‐ATRP strategy could be applied to synthesize brush polymers via grafting from linear polymers containing aromatic bromide groups. Poly(bromo‐styrene) (PBS, 6.7 kDa, *Đ* = 1.42) synthesized through free radical polymerization, was employed as macroinitiator. Under the established condition, the grafting polymerization of St exhibited a pseudo‐first‐order kinetics (Figure 5a). The resulting brush PS‐*g*‐PS, was successfully prepared (*M*_n _= 41.9 kDa, *Đ* = 1.39) as supported by the shift in SEC traces (Figure 5b) and ^1^H NMR spectra (Figure S37).

**Figure 5:**
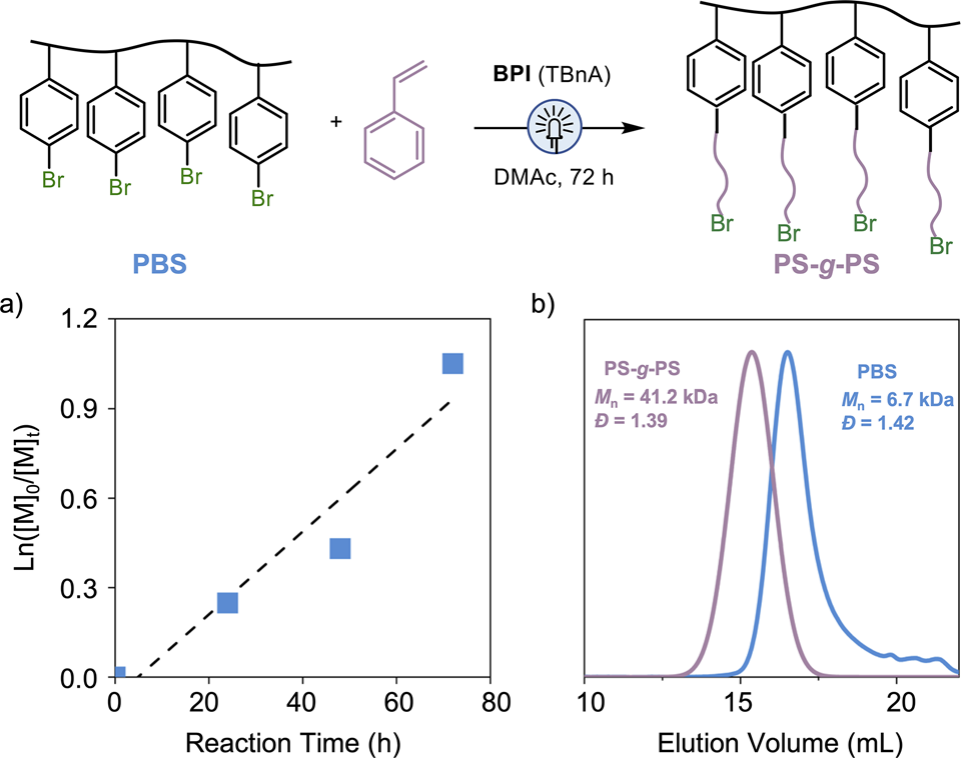
The “grafting from” process via O‐ATRP of St from poly(4‐bromostyrene) (PBS) synthesized by free radical polymerization. [St]:[Ar‐Br]:[**BPI**]:[TBnA] = 100:1:0.01:5 in DMAc, 450 nm LED.

In this work, we employed a super‐reducing PC system toward the O‐ATRP of monomers and initiators that have been challenging in O‐ATRP. This system offered polymerization control, air tolerance, and temporal regulation. The expanded capability of this system was demonstrated with a wide range of initiators. Looking ahead, the mechanistic insights gained here will guide the development of more user‐friendly O‐ATRP platforms.

## Conflict of Interests

The authors declare no conflict of interest.

## Supporting information



Supporting Information

## Data Availability

The data that support the findings of this study are available in the Supporting Information of this article.
