# Supplementary material for: Organocatalyzed Atom Transfer Radical Polymerization (O‐ATRP) Using a Super‐Reducing Photoredox Catalyst
Source: Angew Chem Int Ed Engl. 2025 Oct 8;64(47):e202517641. doi: 10.1002/anie.202517641 (PMC12624307; doi:10.1002/anie.202517641)
Supplement: Supplementary file 1 — Supporting Information [file ANIE-64-e202517641-s001.pdf]

SUPPORTING INFORMATION

Organocatalyzed Atom Transfer Radical Polymerization (O-ATRP) Using a Super-Reducing Photoredox Catalyst

Yucheng Zhao,[a] Brandon S. Portela,[a] Alexander R. Green,[a] Anna M. Wolff,[a] Xin Liu,[a] Katherine O. Puffer,[a] Arindam Sau,[b] Niels H. Damrauer,[b] Robert S. Paton,[a] and Garret M. Miyake*[a]

[a] Y. Zhao, B. S. Portela, A. R. Green, A. Wolff, X. Liu, K. O. Puffer, R. S. Paton, and G. M. Miyake
Department of Chemistry
Colorado State University
Fort Collins, CO 80523, USA.
E-mail: Garret.Miyake@colostate.edu

[b] A. Sau and N. H. Damrauer,
Renewable and Sustainable Energy Institute (RASEI)
Department of Chemistry
University of Colorado Boulder
Boulder, CO, 80309, USA.

Table of Contents

1. General Information of Materials and Analytical Methods 2
2. Exploring O-ATRP with super-reducing photocatalysts: St as the model monomer 2
2.1 General procedures for the O-ATRP of St 2
2.2 Effect of electron donors in O-ATRP of St using BPI as PC 3
2.3 O-ATRP of St using different PC. 4
3. O-ATRP results in Table 1. 7
4. O-ATRP results in Table 2. 11
5. O-ATRP results in Figure 3. 13
6.Grafting from poly(bromostyrene). 16
References 17

1. General Information of Materials and Analytical Methods

1.1 Materials

Styrene (St), methyl methacrylate (MMA), methyl acrylate (MA), acryloyl morpholine (ACMO), vinyl acetate (VAc), ethyl α-bromophenylacetate (EBP), methyl α-bromoisobutyrate (DBMM), methyl bromopropionate (MBP), ethyl α-chlorophenylacetate (ECP), 4-bromobenzotrifluoride (BTFB), 4-chlorobenzotrifluoride, 1-bromoethyl)benzene(PEBr), benzyl chloride (BnC), 1-isothiocyanatoethylbenzene (BESCN), N,N,N',N'',N''-Pentamethyldiethylenetriamine (PMDETA), triethylamine (TEA), N,N-Diisopropylethylamine (DIPEA), diisopropylamine (DIPA), tributylamine (TBA), triisobutylamine (TIBA), 2,2,6,6-tetramethylpiperidine (TMP), 1,2,2,6,6-pentamethylpiperidine (PMP), hantzsch ester (HE), and tribenzylamine (TBnA) were purchased from Sigma-Aldrich. 4-Fluorostyrene (4FSt), 4-chlorostyrene (4CSt) and vinyl carbazole (VCz) were purchased from Oakwood Chemical. Anhydrous N'N''-dimethylformamide (DMF), and N'N''-dimethylacetamide (DMAc), methanol, and other solvents were purchased from Sigma Aldrich or Fisher. N'N''-dimethylacetamide (DMAc) was obtained and purified using an mBraun MB-SPS-800 solvent purification system and kept under a nitrogen atmosphere. All monomers and initiators were distilled under vacuum or passed through an activated basic alumina column to remove inhibitor, then transferred to amber vials in a nitrogen filled glove box before use. Benzo[ghi]perylene monoamide (**BPI**) derivatives were synthesized following established literature protocols.[1]

1.2 Analytical methods

Nuclear magnetic resonance (NMR) spectra were obtained using a Bruker 400 MHz NMR Spectrometer at 298 K. All ^{1}H NMR experiments are reported in parts per million (ppm) and were measured relative to the signals for residual chloroform (δ = 7.26 ppm) in deuterated chloroform. High resolution mass spectrometry was performed using a Bruker ultrafleXtreme MALDI-TOF/TOF.

Polymer molecular weight analysis was conducted using size exclusion chromatography (SEC) coupled with multi-angle light scattering (MALS). The system consisted of an Agilent HPLC equipped with a guard column and three PLgel 5 μm MIXED-C gel permeation columns. Detection was performed using a Wyatt Technology TrEX differential refractometer and a Wyatt Technology miniDAWN TREOS light scattering detector. Tetrahydrofuran (THF) was used as the eluent at a flow rate of 1.0 mL/min at 25 °C. A refractive index increment (*d*n/*d*c) value of 0.184 was applied for the molecular weight analysis of PS.

Steady state fluorescence spectroscopy was performed using a FS5 Spectrofluorometer from Edinburgh Instruments. TCSPC was performed using an FS5 Spectrofluorometer from Edinburgh Instruments with a TCSPC upgrade and a 365 nm EPLED from Edinburgh Instruments. Lifetimes were determined by tail-fitting. UV-Visible spectroscopy was performed using an Agilent Cary 5000.

Matrix-assisted laser desorption/ionization (MALDI) mass spectrometry was performed on a Bruker ultrafleXtreme MALDI-TOF/TOF instrument. A polymer stock solution was prepared at 5 mg/mL in THF. For spotting on the MALDI target plate, the mixture contained 2 μL of the polymer stock solution (5.0 mg/mL), 20 μL of a DCTB matrix solution in THF (60 mg/mL; trans-2-[3-(4-tert-butylphenyl)-2-methyl-2-propenylidene]malononitrile), and 1 μL of silver TFA (5.0 mg/mL). A 15 kDa polymethyl methacrylate standard was used as the calibrant.

2. Exploring O-ATRP with super-reducing photocatalysts: St as the model monomer

2.1 General procedures for the O-ATRP of St

In a N_2 filled glove box, to a 10 mL vial with a stir bar was charged with 0.10 mmol donors, 493 μL (8.11×10^{-4} M) solution of PC, EBP (3.50 μL, 20.00 μmol) and 460 μL St (4.00 mmol). Then, the vial was sealed, taken out of glove box, and exposed under 450 nm light for 48 h with stirring. After polymerization, a 50.0 μL aliquot of the reaction was taken and analyzed by ^{1}H NMR to give the conversion. The solution was diluted with 250 μL DCM, precipitated in methanol, and dried under vacuum at 70 °C for 24 h to afford the polymer. The M_n and $Đ$ were analyzed by size exclusion chromatography coupled with multi-angle light scattering.

2.2 Effect of electron donors in O-ATRP of St using BPI as PC

Table S1. Effect of electron donors in the O-ATRP of St

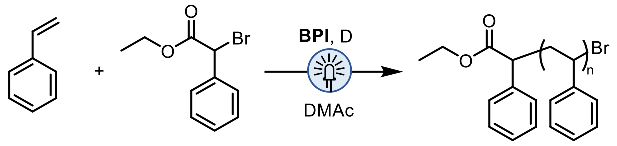

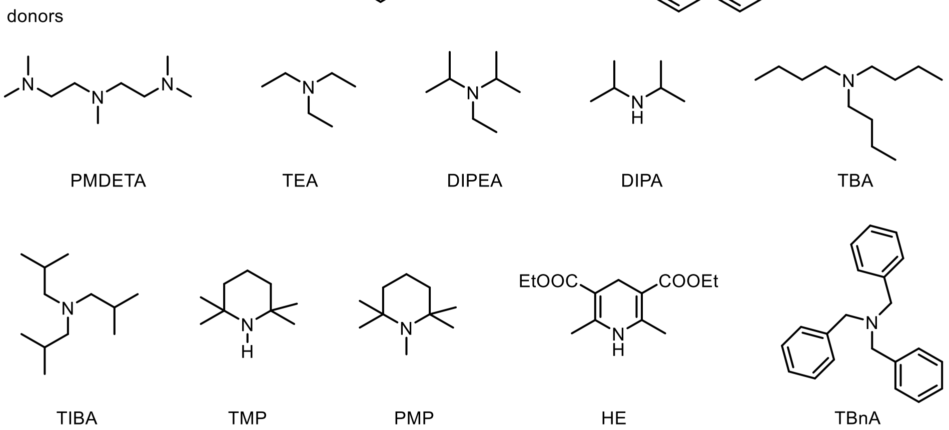

Entry	D	Conv. (%)	$M_{n,theo}$ (kDa)	M_n (kDa)	*Đ*	*I** *(%)*
S1	PMDETA	53	11.1	13.6	1.33	82
S2	DIPEA	63	13.2	12.8	1.48	103
S3	DIPA	66	13.8	9.0	1.25	153
S4	TEA	49	10.2	12.4	1.78	82
S5	TBA	55	11.4	10.2	1.47	112
S6	TIBA	72	15.1	10.0	1.87	151
S7	HE	59	12.4	8.7	1.48	143
S8	TBnA	82	17.3	17.9	1.24	96
S9	PMP	81	16.9	6.8	1.61	248
S10	TMP	61	12.8	6.4	1.69	200
S11	-	51	10.8	37.0	1.36	29
S12	TBnA (0.5 eq)	19	4.1	21.5	1.26	19
S13	TBnA (1 eq)	20	4.3	12.2	1.13	35
S14	TBnA (2 eq)	53	11.1	13.6	1.33	82
S15	TBnA (10 eq)	49	10.3	24.9	1.14	41

[a][St]/[EBP]/[**BPI**]/[D] = 200/1/0.02/5 in DMAc, 450 nm LED light, 25 ºC, ambient pressure, 48 h. Conversions were based on ^{1}H NMR analysis of remaining St after reactions. $M_{n,theo}$ were calculated based on St conversions. M_n and *Đ* were measured by SEC-MALS.

Compared to electron donors that can initiate new chains through their α-amino radicals (entries S1-S2 and S4-S6) or proton donors (entries S3, S7, and S10), TBnA provided better control over polymerization by suppressing side reactions.

2.3 O-ATRP of St using different PC.

Table S2. The O-ATRP of St using different PCs.[a]

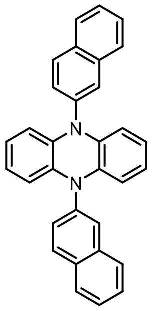
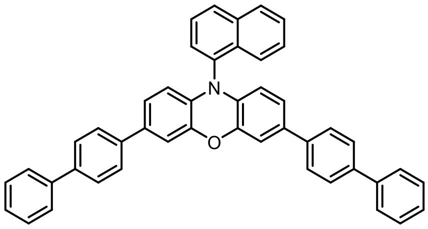
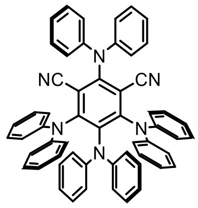
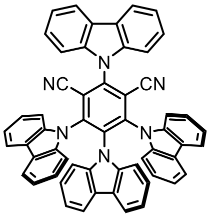

PhenN Phenox 4DPAIPN 4CzIPN

Entry	PC	D	Conv. (%)	$M_{n,theo}$ (kDa)	M_n (kDa)	*Đ*	*I** (%)
S1	PhenN	-	26	5.6	31.2	1.38	18
S2	PhenN	TBnA	12	2.7	30.1	1.47	9.0
S3	Phenox	-	27	5.9	42.1	1.41	14
S4	Phenox	TBnA	37	7.9	56.0	1.18	14
S5	4DPAIPN	-	38	8.1	25.4	1.25	32
S6	4DPAIPN	TBnA	39	8.4	6.4	1.97	131
S7	4CzIPN	-	20	4.4	32.2	1.55	14
S8	4CzIPN	TBnA	25	5.4	35.0	1.43	15
S9	BPI	-	51	10.9	37.1	1.36	29
S10	BPI	TBnA	82	17.3	17.9	1.24	97

[a][St]/[EBP]/[PC]/[TBnA] = 200/1/0.02/5, 450 nm LED light, 25 ºC, 48 h. Conversions were based on ^{1}H NMR analysis of remaining St after reactions. $M_{n,theo}$ were calculated based on St conversions. M_n and *Đ* were measured by SEC-MALS.

Table S3. Calculations of the driving force for electron transfer to a substrate.[a]

PC (D)	$E(PC^{•+}/PC^*)$ (V vs SCE)	$E(PC/PC^{•-})$ (V vs SCE)	ΔG_{ET} (V vs SCE)	k_{app} (hour^{-1})
PhenN	-2.20	-	-0.70	6.3 ×10^{-3}
PhenN (TBnA)	-2.20			2.3×10^{-3}
Phenox	-1.80	-	-0.30	6.8×10^{-3}
Phenox (TBnA)	-1.80			1.0×10^{-2}
4DPAIPN	-1.41	-	0.09	9.3×10^{-3}
4DPAIPN (TBnA)	-	-1.65	-0.15	1.0×10^{-2}
4CzIPN	-1.04	-	0.46	4.4×10^{-3}
4CzIPN (TBnA)	-	-1.21	0.29	5.9×10^{-3}
BPI	-	-1.24		1.4×10^{-2}
**BPI** (TBnA)	-3.64	-	-2.14	3.6×10^{-2}

Note: ΔG_{ET} was estimated using the Rehm-Weller equation:

$$\Delta G = E(D^+/D) - E(A/A^-) - \Delta G_{00} - \frac{e^2}{ed}$$

Where the reduction potential (E_{red}) of PS-Br taken as approximately -1.50 V vs. SCE.[2] A constant value of -0.047 eV was selected, corresponding to a distance of r = 8 Å, which represents an intermediate value within the range previously mentioned.[3]

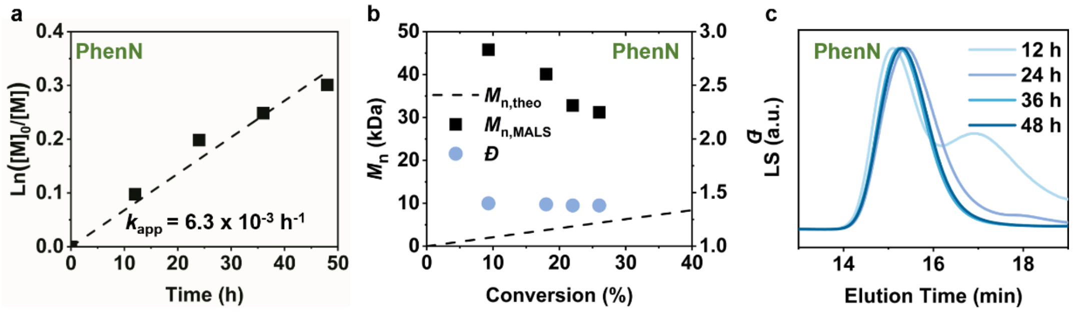

Figure S1. O-ATRP of St under conditions from entry S1 in Table S2, with [St]/[EBP]/[PhenN] = 200/1/0.02. a) Pseudo-first-order kinetics plot. b) Plot showing the evolution of polymer M_n as a function of conversion (black squares) and $Đ$ (blue circles, secondary y-axis) at timepoints of 12, 24, 36, and 48 hours. $M_{n,theo}$ values are indicated by the black dashed line. c) SEC traces of the polymers corresponding to each timepoint.

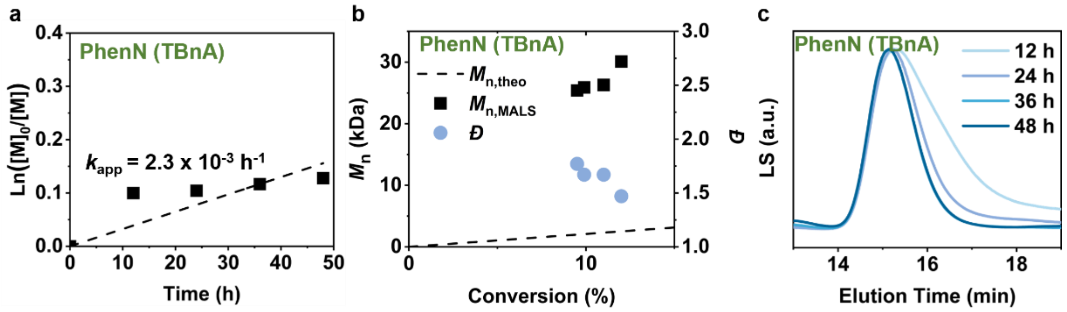

Figure S2. O-ATRP of St under conditions from entry S2 in Table S2, with [St]/[EBP]/[PhenN]/[TBnA] = 200/1/0.02/5. a) Pseudo-first-order kinetics plot. b) Plot showing the evolution of polymer M_n as a function of conversion (black squares) and $Đ$ (blue circles, secondary y-axis) at timepoints of 12, 24, 36, and 48 hours. $M_{n,theo}$ values are indicated by the black dashed line. c) SEC traces of the polymers corresponding to each timepoint.

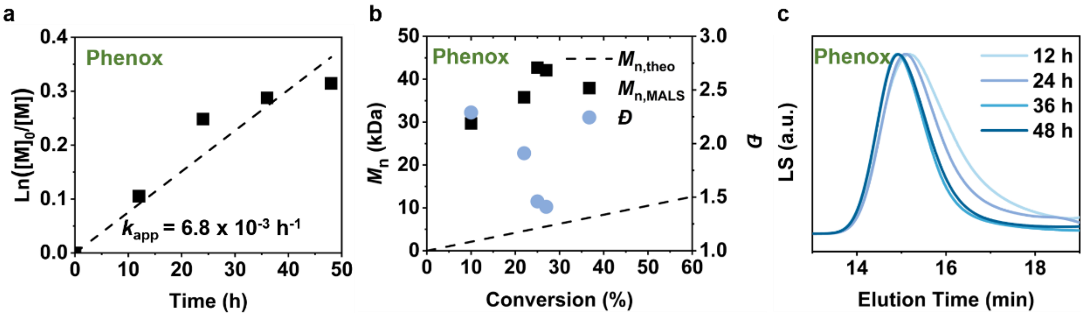

Figure S3. O-ATRP of St under conditions from entry S3 in Table S2, with [St]/[EBP]/[Phenox] = 200/1/0.02. a) Pseudo-first-order kinetics plot. b) Plot showing the evolution of polymer M_n as a function of conversion (black squares) and $Đ$ (blue circles, secondary y-axis) at timepoints of 12, 24, 36, and 48 hours. $M_{n,theo}$ values are indicated by the black dashed line. c) SEC traces of the polymers corresponding to each timepoint.

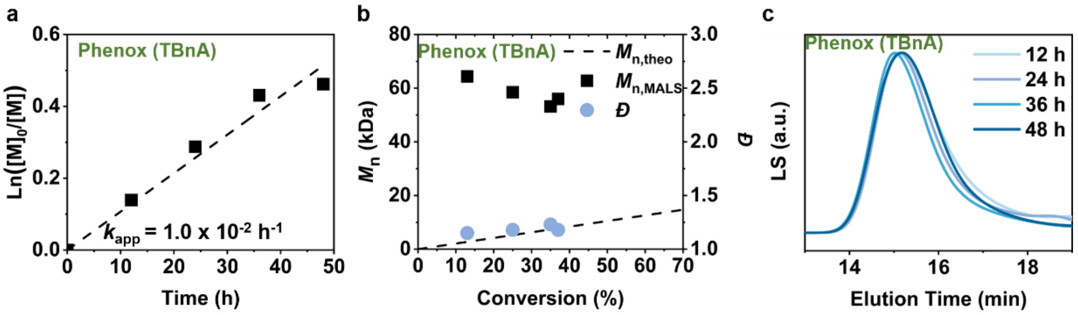

Figure S4. O-ATRP of St under conditions from entry S4 in Table S2, with [St]/[EBP]/[Phenox]/[TBnA] = 200/1/0.02/5. a) Pseudo-first-order kinetics plot. b) Plot showing the evolution of polymer M_n as a function of conversion (black squares) and $Đ$ (blue circles, secondary y-axis) at timepoints of 12, 24, 36, and 48 hours. $M_{n,theo}$ values are indicated by the black dashed line. c) SEC traces of the polymers corresponding to each timepoint.

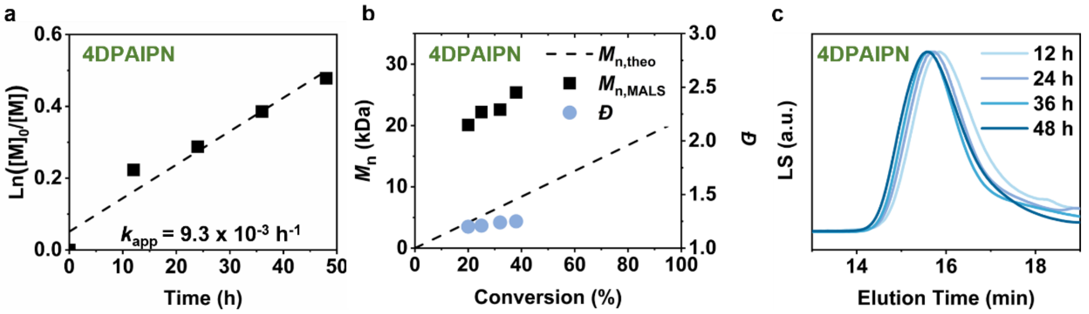

Figure S5. O-ATRP of St under conditions from entry S5 in Table S2, with [St]/[EBP]/[4DPAIPN] = 200/1/0.02. a) Pseudo-first-order kinetics plot. b) Plot showing the evolution of polymer M_n as a function of conversion (black squares) and *Đ* (blue circles, secondary y-axis) at timepoints of 12, 24, 36, and 48 hours. $M_{n,theo}$ values are indicated by the black dashed line. c) SEC traces of the polymers corresponding to each timepoint.

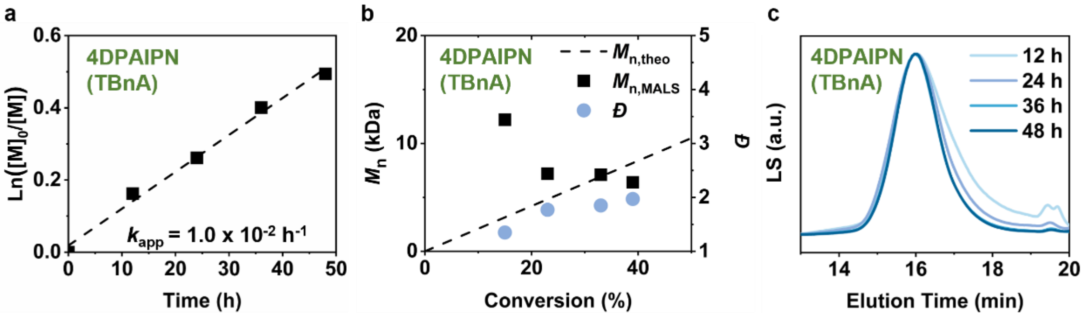

Figure S6. O-ATRP of St under conditions from entry S6 in Table S2, with [St]/[EBP]/[4DPAIPN]/[TBnA] = 200/1/0.02/5. a) Pseudo-first-order kinetics plot. b) Plot showing the evolution of polymer M_n as a function of conversion (black squares) and *Đ* (blue circles, secondary y-axis) at timepoints of 12, 24, 36, and 48 hours. $M_{n,theo}$ values are indicated by the black dashed line. c) SEC traces of the polymers corresponding to each timepoint.

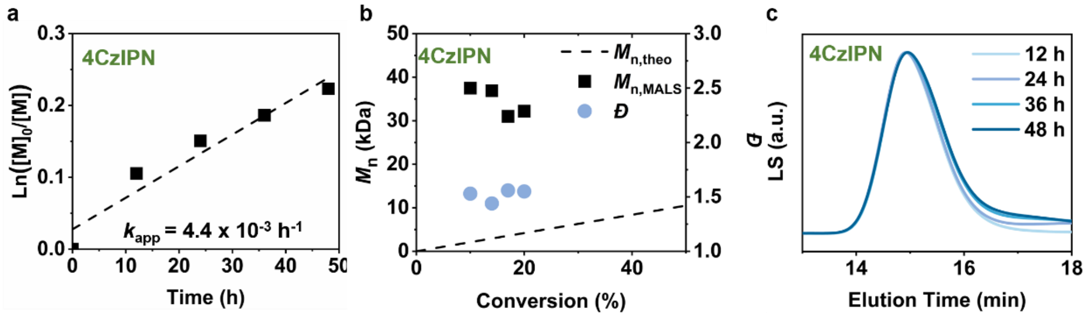

Figure S7. O-ATRP of St under conditions from entry S7 in Table S2, with [St]/[EBP]/[4CzIPN] = 200/1/0.02. a) Pseudo-first-order kinetics plot. b) Plot showing the evolution of polymer M_n as a function of conversion (black squares) and *Đ* (blue circles, secondary y-axis) at timepoints of 12, 24, 36, and 48 hours. $M_{n,theo}$ values are indicated by the black dashed line. c) SEC traces of the polymers corresponding to each timepoint.

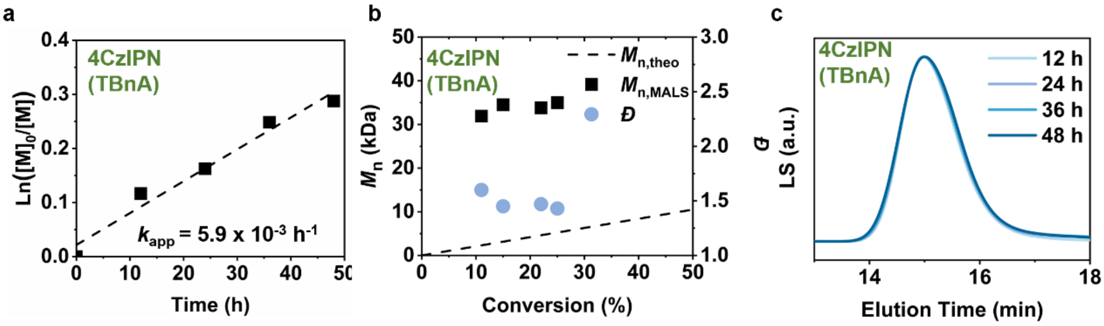

Figure S8. O-ATRP of St under conditions from entry S8 in Table S2, with [St]/[EBP]/[4CzIPN]/[TBnA] = 200/1/0.02/5. a) Pseudo-first-order kinetics plot. b) Plot showing the evolution of polymer M_n as a function of conversion (black squares) and *Đ* (blue circles, secondary y-axis) at timepoints of 12, 24, 36, and 48 hours. $M_{n,theo}$ values are indicated by the black dashed line. c) SEC traces of the polymers corresponding to each timepoint.

3. O-ATRP results in Table 1.

*O-ATRP of St using **BPI** as a catalyst.*

In a N_2 filled glove box, to a 10 mL vial with a stir bar was charged with 0.100 mmol donors, 493 μL (8.11×10^{-4} M) solution of BPI, EBP (3.50 μL, 20.0 μmol) and 460 μL St (4.00 mmol). Then, the vial was sealed, taken out of glove box, and exposed under 450 nm light for 48 h with stirring. At corresponding timepoint, a 50.0 μL aliquot of the reaction was taken and analyzed by ^{1}H NMR to give the conversion. The M_n and $Đ$ were analyzed by size exclusion chromatography coupled with multi-angle light scattering.

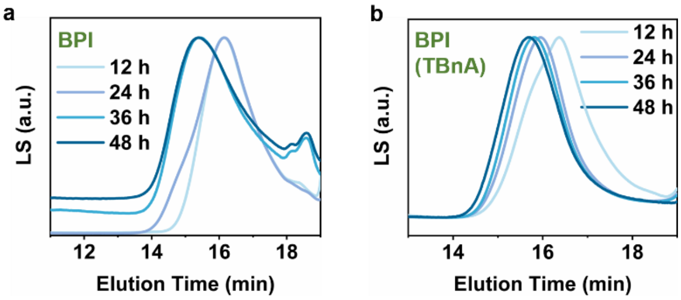

Figure S9. SEC traces of O-ATRP of St using BPI and **BPI** (TBnA). The associated kinetic studies are shown in **Figure 3** of the main text. The k_{app} fits results were listed in **Table S3**.

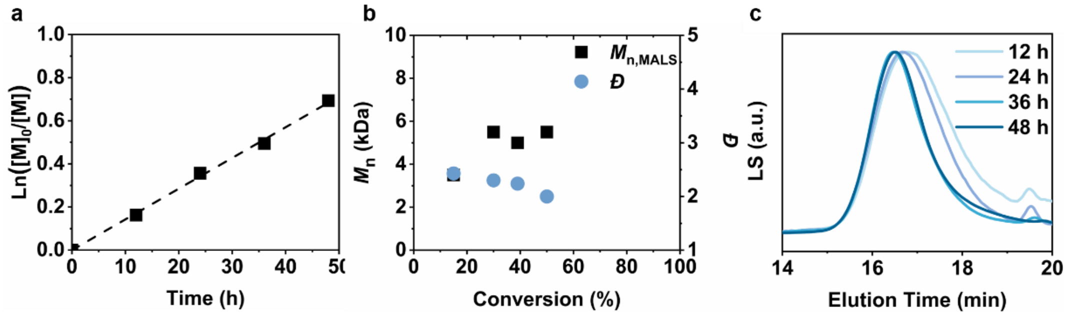

Figure S10. O-ATRP of St under conditions from entry 1 in Table 1, with [St]/[EBP]/[**BPI**] = 200/0/0.02. a) Pseudo-first-order kinetics plot. b) Plot showing the evolution of polymer M_n as a function of conversion (black squares) and $Đ$ (blue circles, secondary y-axis) at timepoints of 12, 24, 36, and 48 hours. c) SEC traces of the polymers corresponding to each timepoint.

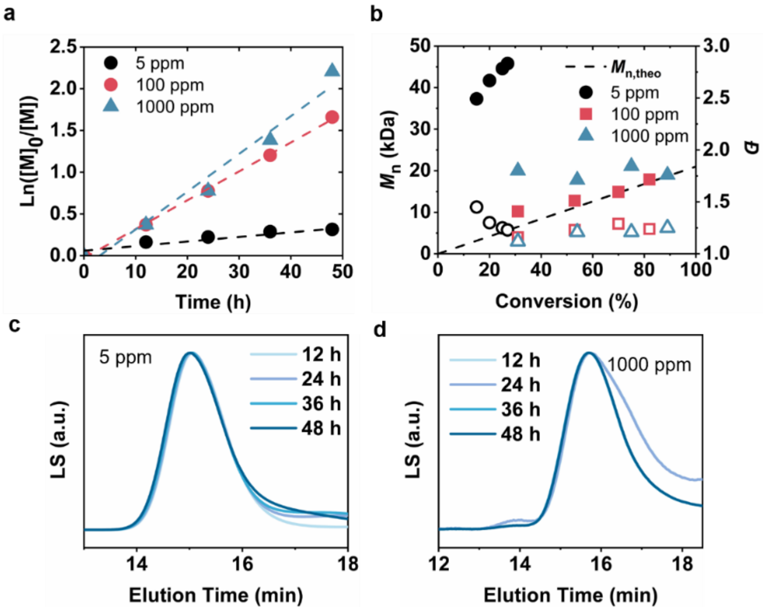

Figure S11. O-ATRP of St using different catalyst loadings (5, 100, and 1000 ppm) for entries 2-4 in Table 1.: a) Pseudo-first-order kinetics plot. b) Plot showing the evolution of polymer M_n as a function of conversion and $Đ$ at timepoints of 12, 24, 36, and 48 hours. $M_{n,theo}$ values are indicated by the black dashed line c) SEC traces of the polymers corresponding to each timepoint using 5 ppm catalyst. d) SEC traces of the polymers corresponding to each timepoint using 1000 ppm catalyst. See Figure S9 to find the SEC traces of the polymers corresponding to each timepoint using 100 ppm catalyst.

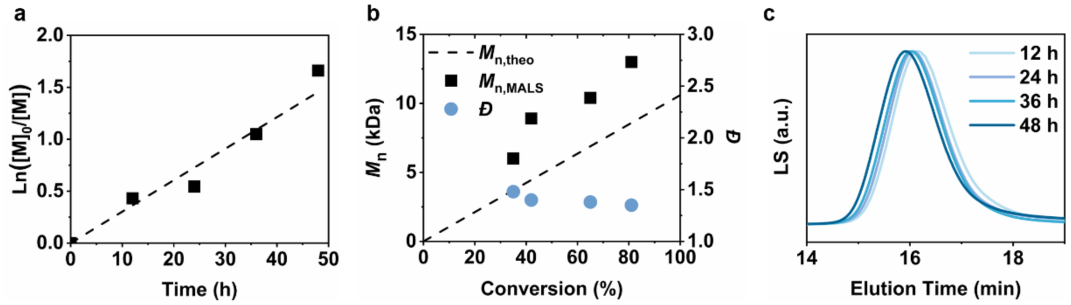

Figure S12. O-ATRP of St with the feed ratio of [St]/[EBP]/[**BPI**] = 100:1:0.01 (entry 5 in Table 1): a) Pseudo-first-order kinetics plot. b) Plot showing the evolution of polymer M_n as a function of conversion and $Đ$ at timepoints of 12, 24, 36, and 48 hours. $M_{n,theo}$ values are indicated by the black dashed line c) SEC traces of the polymers corresponding to each timepoint.

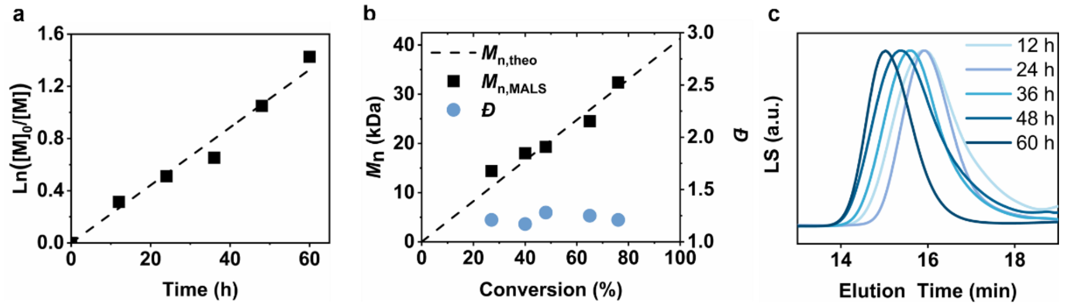

Figure S13. O-ATRP of St with the feed ratio of [St]/[EBP]/[**BPI**] = 400:1:0.04 (entry 6 in Table 1): a) Pseudo-first-order kinetics plot. b) Plot showing the evolution of polymer M_n as a function of conversion and $Đ$ at timepoints of 12, 24, 36, 48 and 60 hours. $M_{n,theo}$ values are indicated by the black dashed line c) SEC traces of the polymers corresponding to each timepoint.

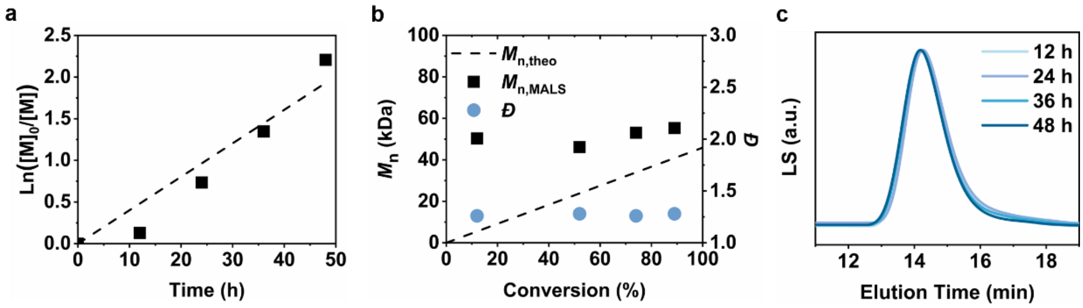

Figure S14. O-ATRP of St with the feed ratio of [St]/[EBP]/[**BPI**] = 400:1:0.04 at 50 ºC. a) Pseudo-first-order kinetics plot. b) Plot showing the evolution of polymer M_n as a function of conversion and $Đ$ at timepoints of 12, 24, 36, and 48 hours. $M_{n,theo}$ values are indicated by the black dashed line c) SEC traces of the polymers corresponding to each timepoint.

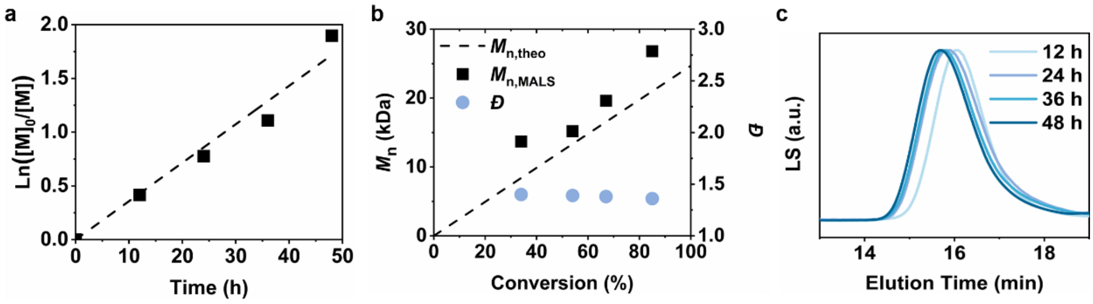

Figure S15. O-ATRP of 4FSt with the feed ratio of [4FSt]/[EBP]/[**BPI**]= 200:1:0.02 (entry 7 in Table 1): a) Pseudo-first-order kinetics plot. b) Plot showing the evolution of polymer M_n as a function of conversion and $Đ$ at timepoints of 12, 24, 36, and 48 hours. $M_{n,theo}$ values are indicated by the black dashed line c) SEC traces of the polymers corresponding to each timepoint.

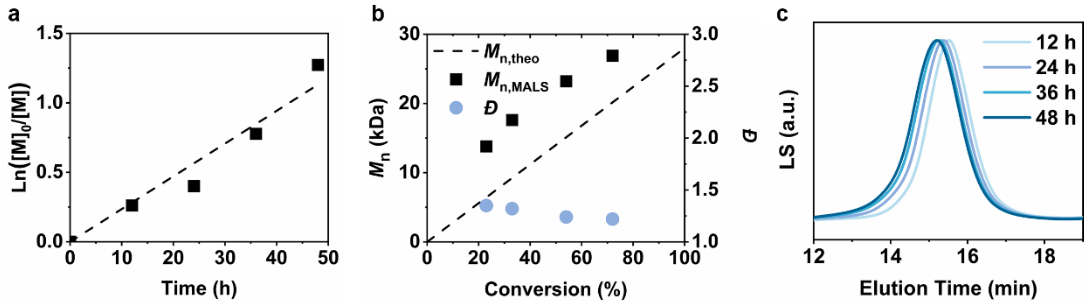

Figure S16. O-ATRP of 4CSt with the feed ratio of [4CSt]/[EBP]/[**BPI**]= 200:1:0.02 (entry 8 in Table 1): a) Pseudo-first-order kinetics plot. b) Plot showing the evolution of polymer M_n as a function of conversion and $Đ$ at timepoints of 12, 24, 36, and 48 hours. $M_{n,theo}$ values are indicated by the black dashed line c) SEC traces of the polymers corresponding to each timepoint.

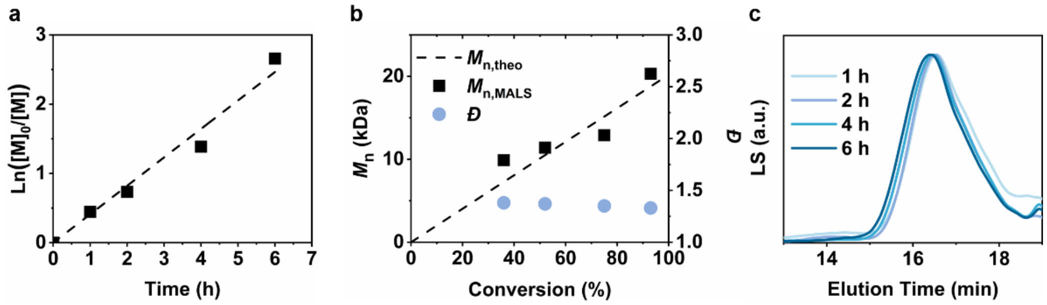

Figure S17. O-ATRP of MMA with the feed ratio of [MMA]/[EBP]/[**BPI**]= 200:1:0.02 (entry 9 in Table 1): a) Pseudo-first-order kinetics plot. b) Plot showing the evolution of polymer M_n as a function of conversion and $Đ$ at timepoints of 1, 2, 4, and 6 hours. $M_{n,theo}$ values are indicated by the black dashed line c) SEC traces of the polymers corresponding to each timepoint.

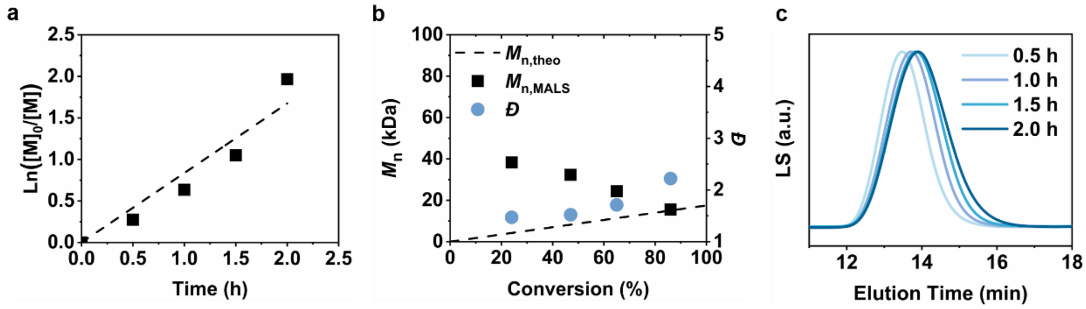

Figure S18. O-ATRP of MA with the feed ratio of [MA]/[EBP]/[**BPI**]= 200:1:0.02 (entry 11 in Table 1): a) Pseudo-first-order kinetics plot. b) Plot showing the evolution of polymer M_n as a function of conversion and $Đ$ at timepoints of 0.5, 1.0, 1.5, and 2.0 hours. $M_{n,theo}$ values are indicated by the black dashed line c) SEC traces of the polymers corresponding to each timepoint.

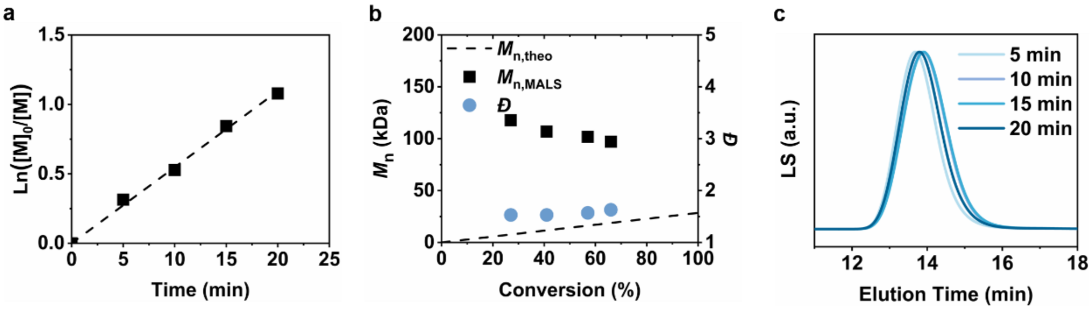

Figure S19. O-ATRP of ACMO with the feed ratio of [ACMO]/[EBP]/[**BPI**]= 200:1:0.02: a) Pseudo-first-order kinetics plot. b) Plot showing the evolution of polymer M_n as a function of conversion and $Đ$ at timepoints of 5, 10, 15, and 20 min. $M_{n,theo}$ values are indicated by the black dashed line c) SEC traces of the polymers corresponding to each timepoint. Due to the high viscosity of the solution, the polymerization was terminated at 20 min.

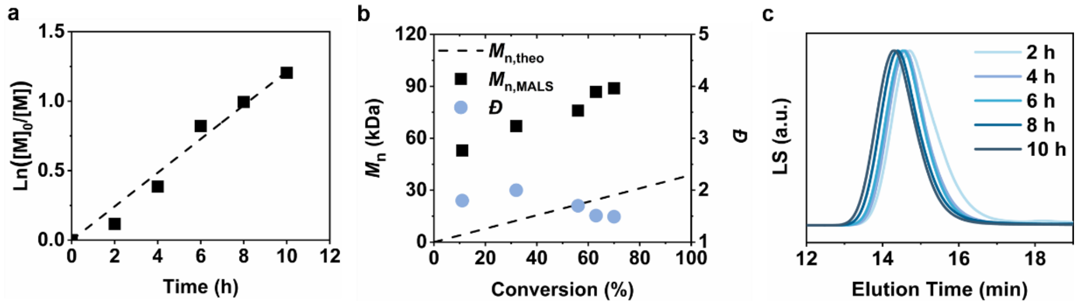

Figure S20. O-ATRP of VCz with the feed ratio of [VCz]/[EBP]/[**BPI**]= 200:1:0.02 (entry 10 in Table 1): a) Pseudo-first-order kinetics plot. b) Plot showing the evolution of polymer M_n as a function of conversion and *Đ* at timepoints of 2, 4, 6, 8, and 10 hours. $M_{n,theo}$ values are indicated by the black dashed line c) SEC traces of the polymers corresponding to each timepoint.

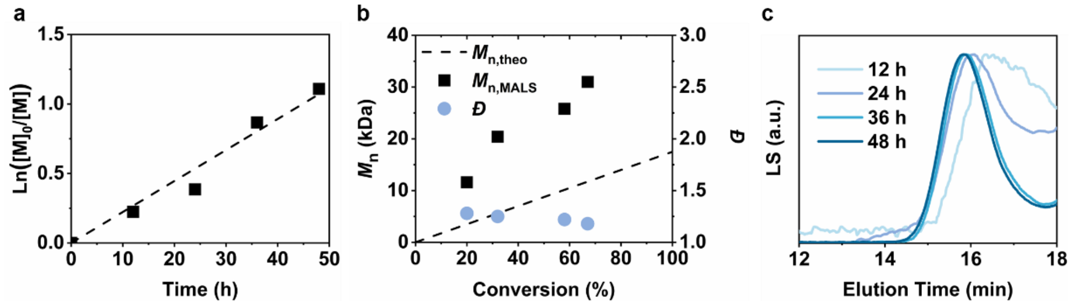

Figure S21. O-ATRP of VAc with the feed ratio of [VAc]/[EBP]/[**BPI**]= 200:1:0.02 (entry 11 in Table 1): a) Pseudo-first-order kinetics plot. b) Plot showing the evolution of polymer M_n as a function of conversion and *Đ* at timepoints of 12, 24, 36, and 48 hours. $M_{n,theo}$ values are indicated by the black dashed line c) SEC traces of the polymers corresponding to each timepoint.

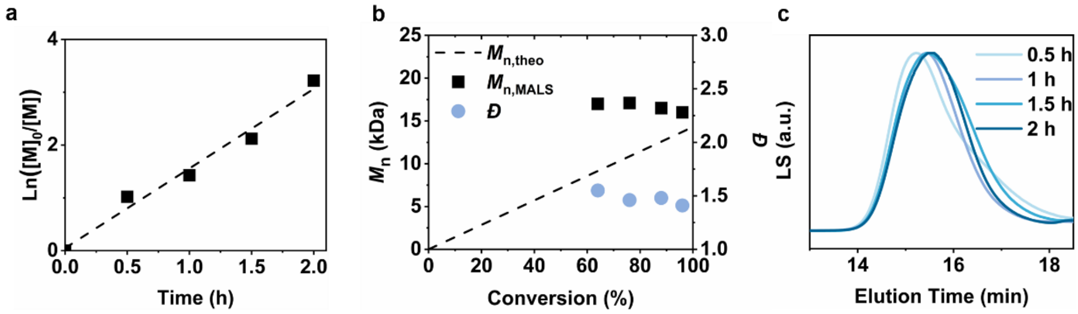

Figure S22. O-ATRP of methyl vinyl ketone (MVK) with the feed ratio of [MVK/[EBP]/[**BPI**]= 200:1:0.02: a) Pseudo-first-order kinetics plot. b) Plot showing the evolution of polymer M_n as a function of conversion and *Đ* at timepoints of 0.5, 1.0, 1.5, and 2.0 hours. $M_{n,theo}$ values are indicated by the black dashed line c) SEC traces of the polymers corresponding to each timepoint. Based on the results, MVK did not exhibit controlled radical polymerization in this system, likely due to its fast propagation rate and highly reactive radical species.

4. O-ATRP results in Table 2.

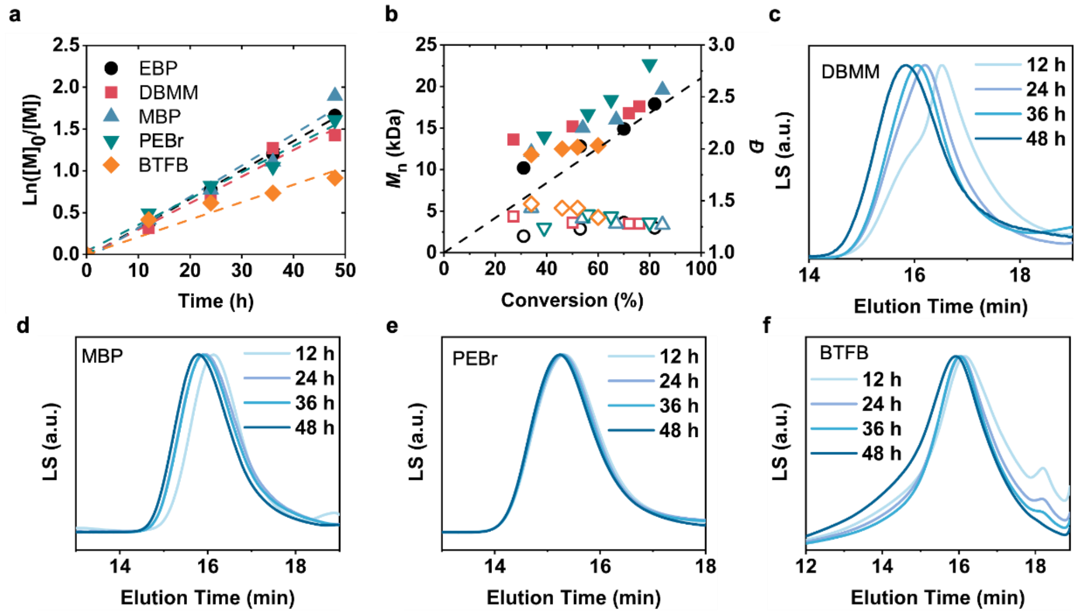

Figure S23. O-ATRP of St using alkyl/aromatic bromides as initiators: a) Pseudo-first-order kinetics plot. b) Plot showing the evolution of polymer M_n as a function of conversion and $Đ$ at timepoints of 12, 24, 36, and 48 hours. $M_{n,theo}$ values are indicated by the black dashed line. c)-f) SEC traces of the polymers corresponding to each timepoint.

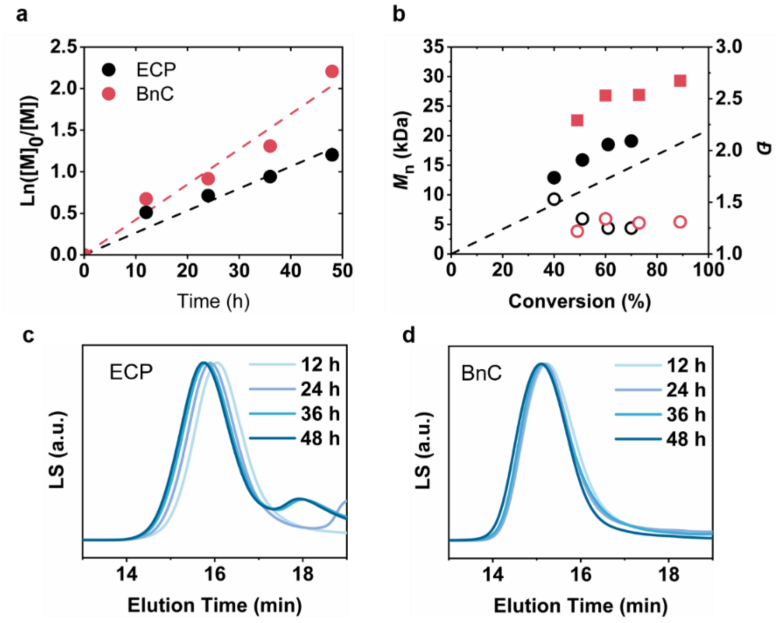

Figure S24. O-ATRP of St using alkyl chlorides as initiators: a) Pseudo-first-order kinetics plot. b) Plot showing the evolution of polymer M_n as a function of conversion and $Đ$ at timepoints of 12, 24, 36, and 48 hours. $M_{n,theo}$ values are indicated by the black dashed line. c)-d) SEC traces of the polymers corresponding to each timepoint.

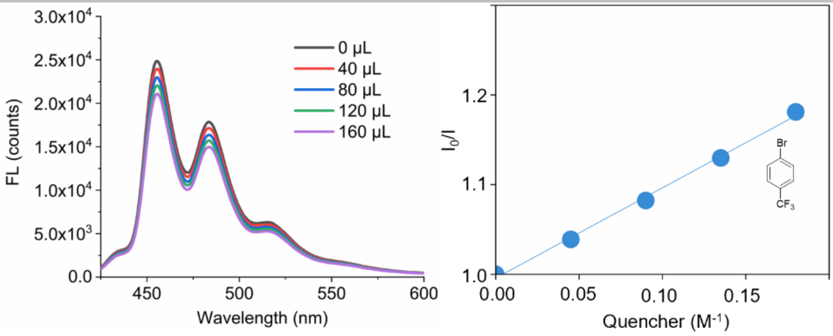

[BFTB] (M^{-1})	I/I_0
0	1
0.045	1.03894
0.09	1.08224
0.135	1.12968
0.18	1.18105

Figure S25. Quenching experiments of BTFB: **BPI** (10^{-5} M) and TBnA (2.5×10^{-4} M) in DMAc solution was subjected to varying concentrations of the quencher (BTFB).

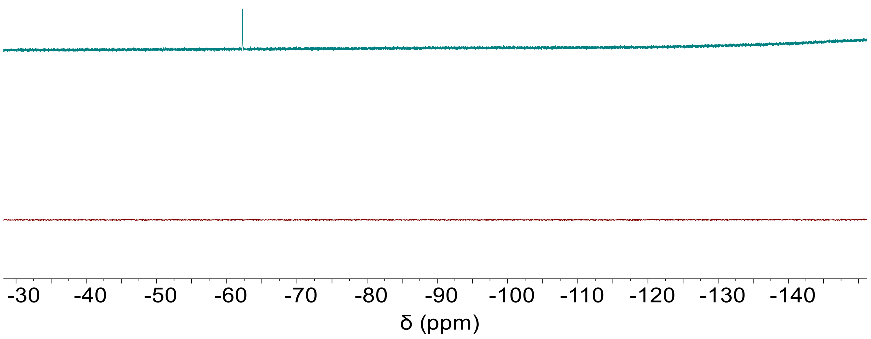

Figure S26. ^{19}F NMR spectra (376 MHz, $CDCl_3$) of PS synthesized using BTFB (top) and the 1-chloro-4-(trifluoromethyl)benzene (bottom).

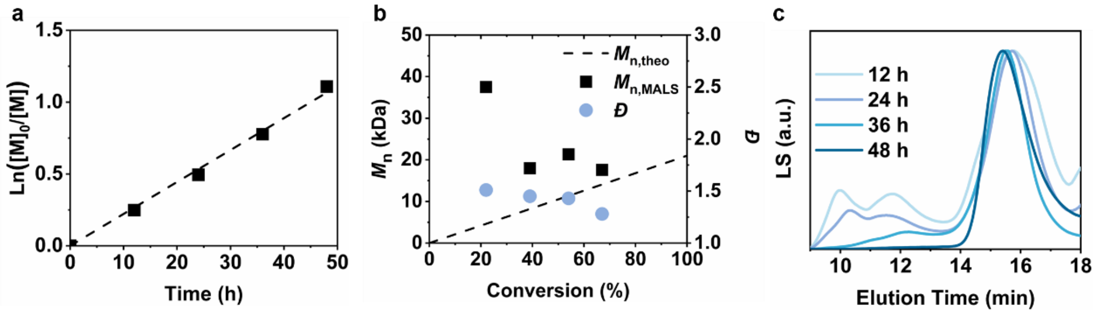

Figure S27. O-ATRP of St using aromatic chloride (1-chloro-4-(trifluoromethyl)benzene) as the initiator: a) Pseudo-first-order kinetics plot. b) Plot showing the evolution of polymer M_n as a function of conversion and $Đ$ at timepoints of 12, 24, 36, and 48 hours. $M_{n,theo}$ values are indicated by the black dashed line. c) SEC traces of the polymers corresponding to each timepoint.

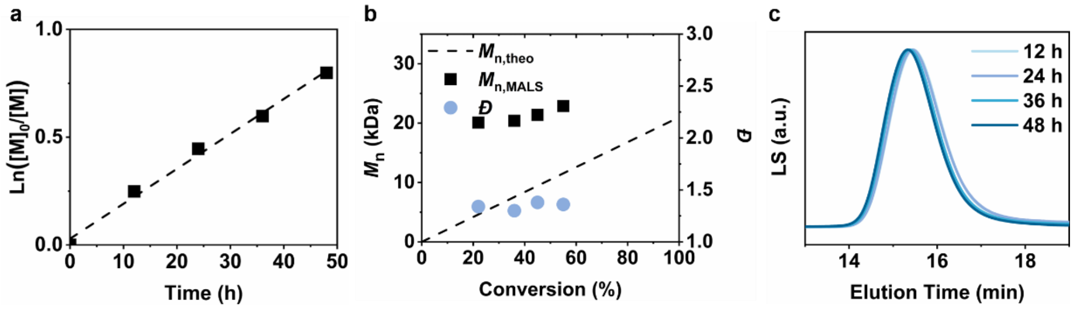

Figure S28. O-ATRP of St using pseudo halide (BESCN) as the initiator: a) Pseudo-first-order kinetics plot. b) Plot showing the evolution of polymer M_n as a function of conversion and $Đ$ at timepoints of 12, 24, 36, and 48 hours. $M_{n,theo}$ values are indicated by the black dashed line. c) SEC traces of the polymers corresponding to each timepoint.

5. O-ATRP results in Figure 3.

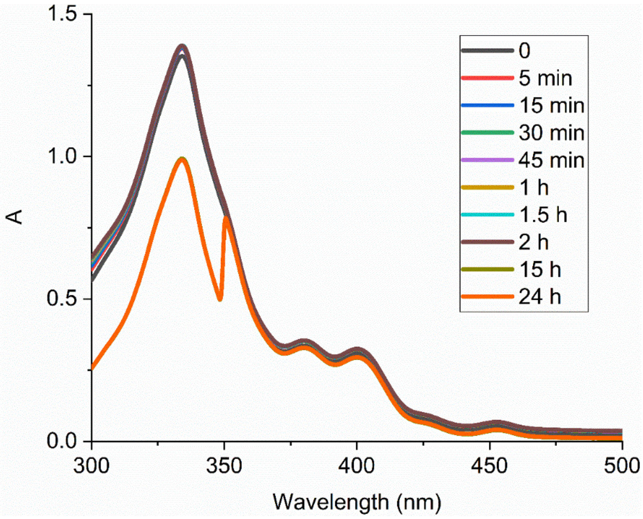

Figure S29. UV-vis spectra of **BPI** (10^{-5} M) and TBnA (2.5×10^{-4} M) in DMAc after 24 hours of exposure to air.

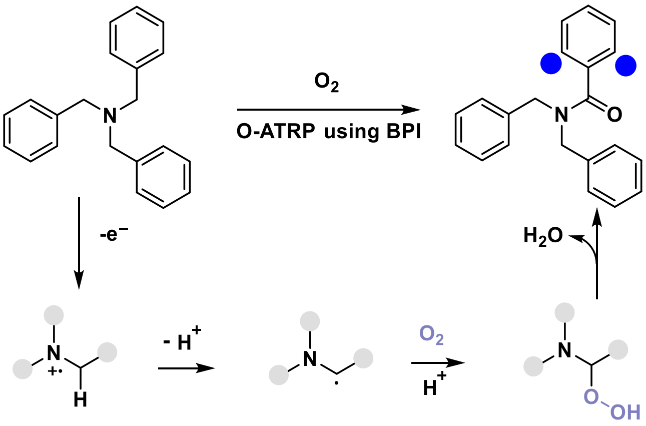

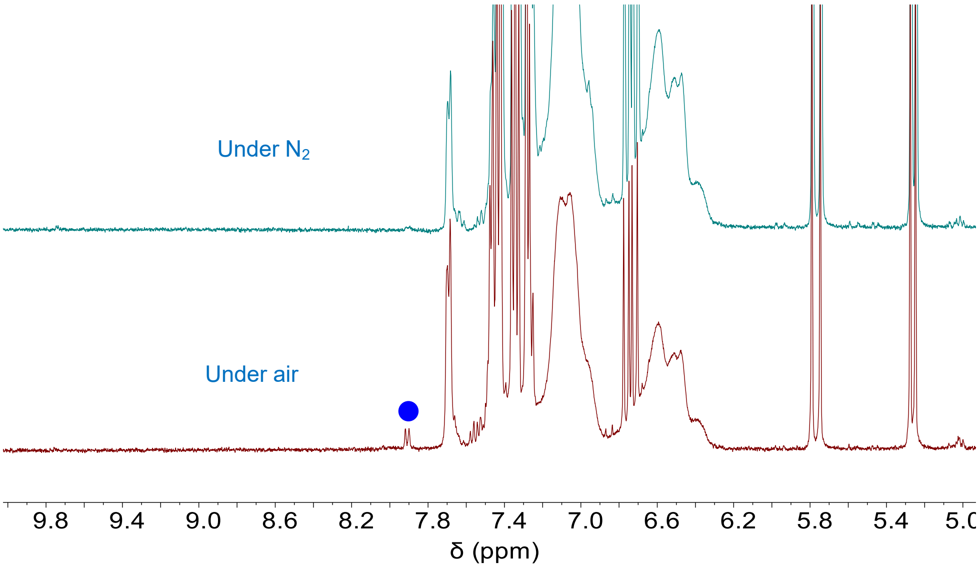

Figure S30. ^{1}H NMR spectra (400 MHz, $CDCl_3$) of St polymerization mixtures collected at the 24-hour timepoint under the conditions described in Figure 3.

For the α-amino radical, it can participate in downstream reactions:

a) halogen atom transfer (XAT) and activation an initiator R-X. The deactivated amine-X decomposes into iminium cation, which can react with nucleophiles.

b) Polymerization can also be initiated by monomer addition. It has been demonstrated that XAT reactivity can be tuned by modifying the α-amino radicals.[4] The α-amino radical derived from TEA reactive to olefin acceptors (k = 2.0 x 10^7 M^{-1} s^{-1}), whereas the α-amino radical from TBnA has been reported to be essentially unreactive (k ~ 10^{-1} M^{-1} s^{-1}). These findings support our results, where TBnA as the electron donor provided better control over polymerization compared to TEA.

c) Reaction with oxygen and formation of amides.[5] In the ^{1}H NMR spectra of O-ATRP conducted under air, we observed the formation of amides, whereas no amides were detected under nitrogen (Figure S30). These results suggest that trapping of O_2 by α-amino radicals may contribute to the air tolerance of this O-ATRP system.

d) For the reduction of radicals, it is generally possible that strongly reducing photocatalysts (PCs) convert radicals into the corresponding carbanions, and this is not limited to extremely reducing PCs. Most reducing PCs are capable of reducing these monomer-derived radicals (E_{red} = –0.63 to –1.53 V vs SCE). However, in RDRP systems such as O-ATRP, the radical concentration is typically very low, which minimizes such side reactions.[6] In our system, we observed that a high catalyst loading (1000 ppm) led to uncontrolled polymerization, likely due to radical-to-carbanion reduction ($E_{red,St\ radical}$ = -1.53 V vs SCE) and direct monomer reduction ($E_{red,St}$ = -2.57 V vs SCE).[6] In contrast, when the catalyst loading was lowered to 100 or 50 ppm, both kinetic studies and molecular weight analysis indicated controlled polymerization (Figure S11), suggesting that these side reactions were effectively suppressed.

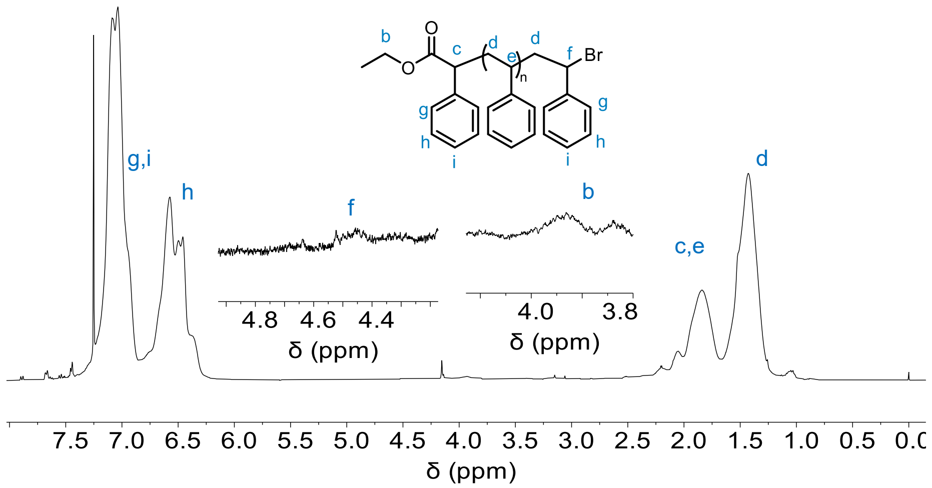

Figure S31. ^{1}H NMR spectra of the PS (400 MHz, $CDCl_3$).

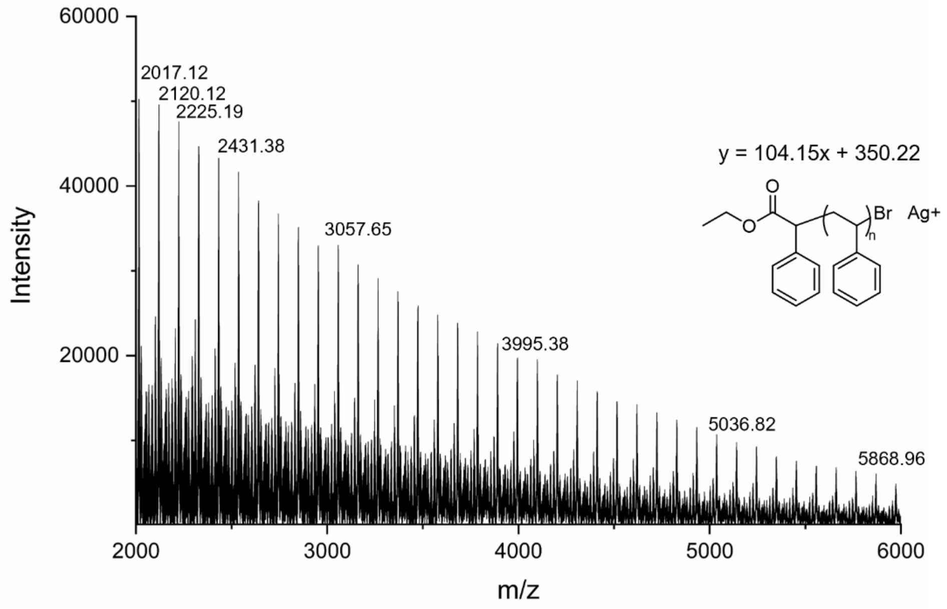

Figure S32. MALDI-TOF mass spectrum of a polystyrene sample.

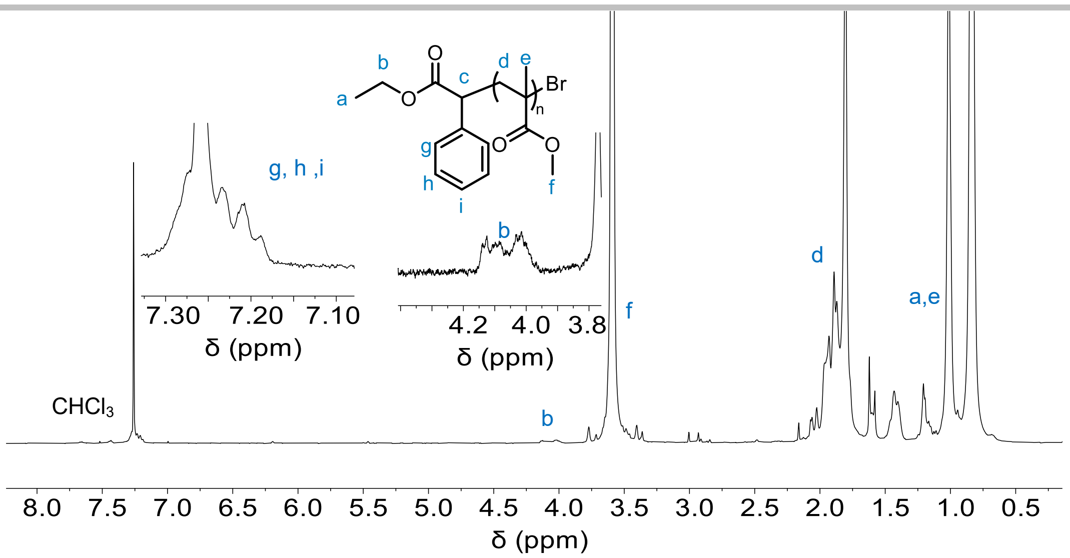

Figure S33. ^{1}H NMR spectra of the PMMA (400 MHz, CDCl$_3$).

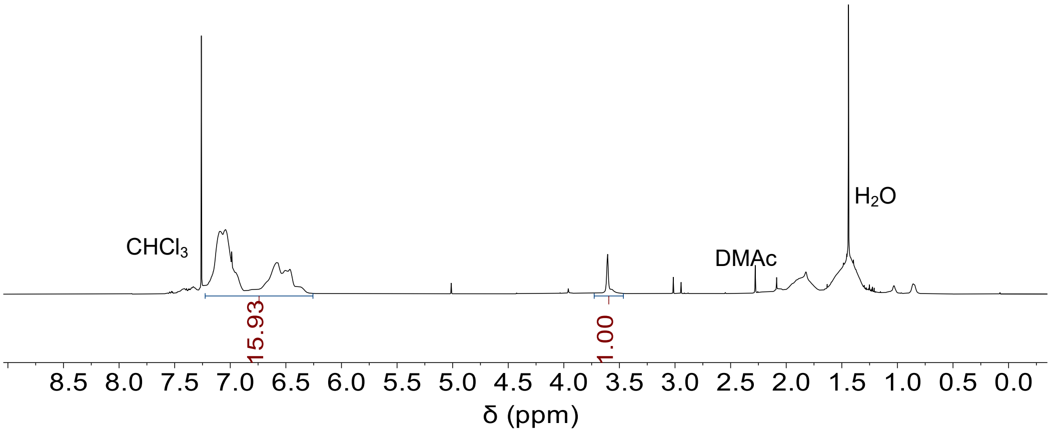

Figure S34. ^{1}H NMR spectrum of the PMMA-*b*-PS (400 MHz, CDCl$_3$).

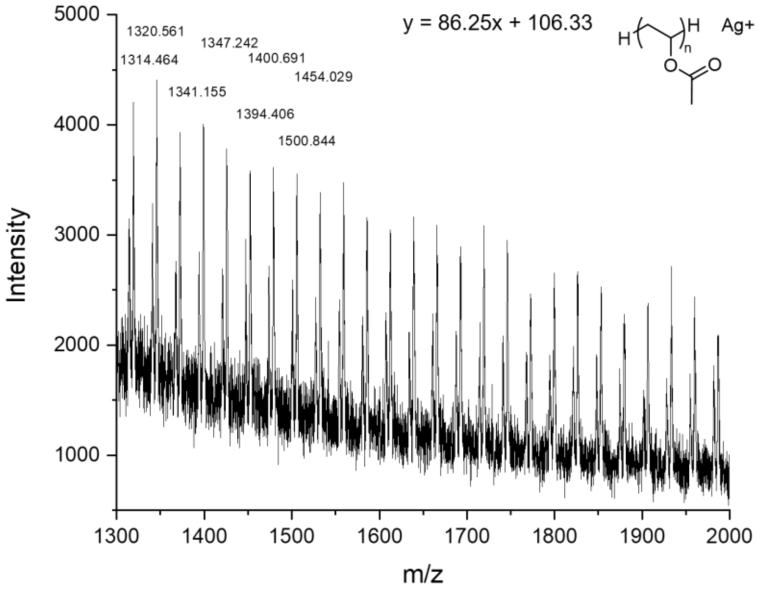

Figure S35. MALDI-TOF mass spectrum of a polyvinyl acetate sample. The principal peaks are assigned a polyvinyl acetate without distinct chain-end groups, whereas the additional signals suggest the presence of more complex species generated during polymerization.

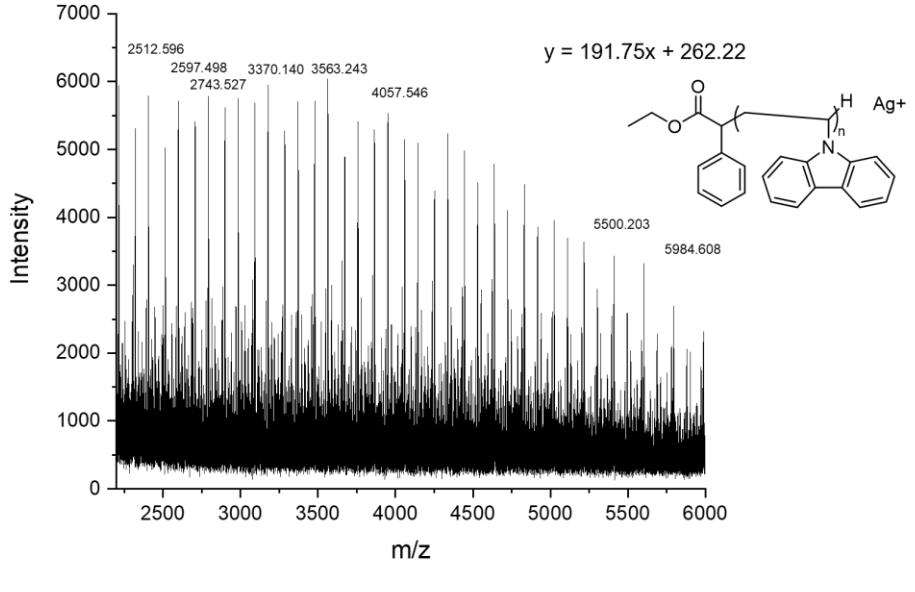

Figure S36. MALDI-TOF mass spectrum of a polyvinyl carbazole sample. The dominant peaks correspond to polymer chains bearing the initiator and a proton as terminal groups, consistent either with Pn–Br halogen loss during analysis or with hydrogen atom transfer (HAT) occurring during polymerization.

6.Grafting from poly(bromostyrene).

Synthesis of poly(bromostyrene) (PBS):

In a 50 mL Schlenk flask, 3.0 g of bromostyrene, 30.0 mg of AIBN, and 10 mL of DMAc were combined. The mixture was sparged with nitrogen for 15 minutes before being placed in a 65 °C oil bath and stirred for 24 hours. Once cooled to room temperature, the reaction mixture was precipitated into methanol to yield PBS. The product was then dried under vacuum at 100 °C for 24 hours and analyzed by SEC, resulting in 1.6 g (53% yield) PBS with M_n = 6.7 kDa, $Đ$ = 1.42.

Grafting from procedure:

In a N_2 filled glove box, to a 10 mL vial with a stir bar was charged with 3.7 mg PBS (20.00 μmol aromatic bromides), 28.7 mg TBnA (0.10 mmol), 246 μL (8.11×10^{-4} M) solution of **BPI**/TBnA, and 2.00 mmol monomer. Then, the vial was sealed, taken out of glove box,

and exposed under 450 nm light with stirring. At corresponding timepoint, a 50.0 µL aliquot of the reaction was taken and analyzed by ^{1}H NMR to give the conversion. The M_n and $Đ$ were analyzed by size exclusion chromatography coupled with multi-angle light scattering.

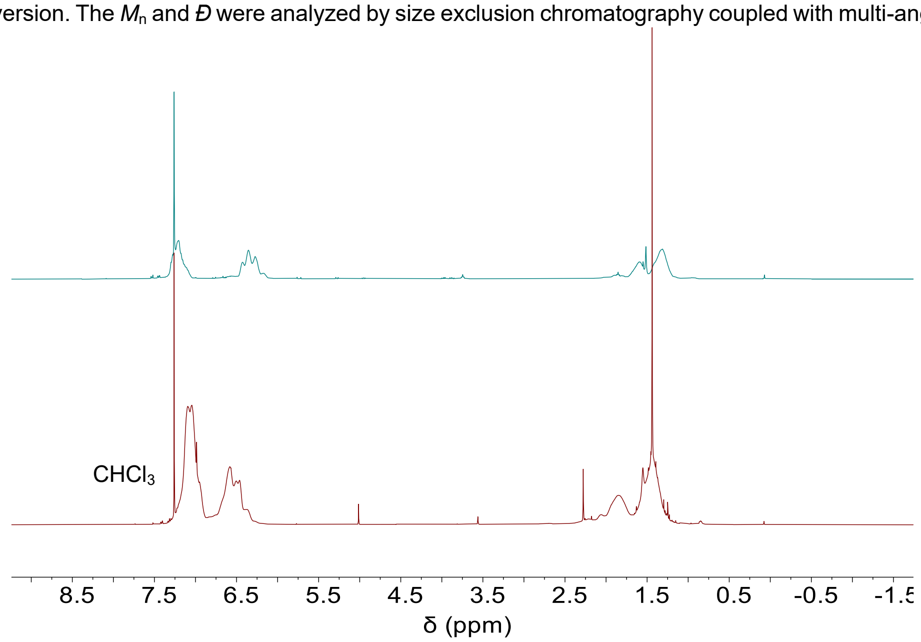

Figure S37. ^{1}H NMR spectra of PBS obtained via free radical polymerization (top) and PBS-*g*-PS synthesized through the grafting-from process for 72 h (400 MHz, $CDCl_3$). The disappearance of peaks in the 6.0–6.5 ppm range, corresponding to aromatic bromides, confirms the complete activation of PBS after grafting.

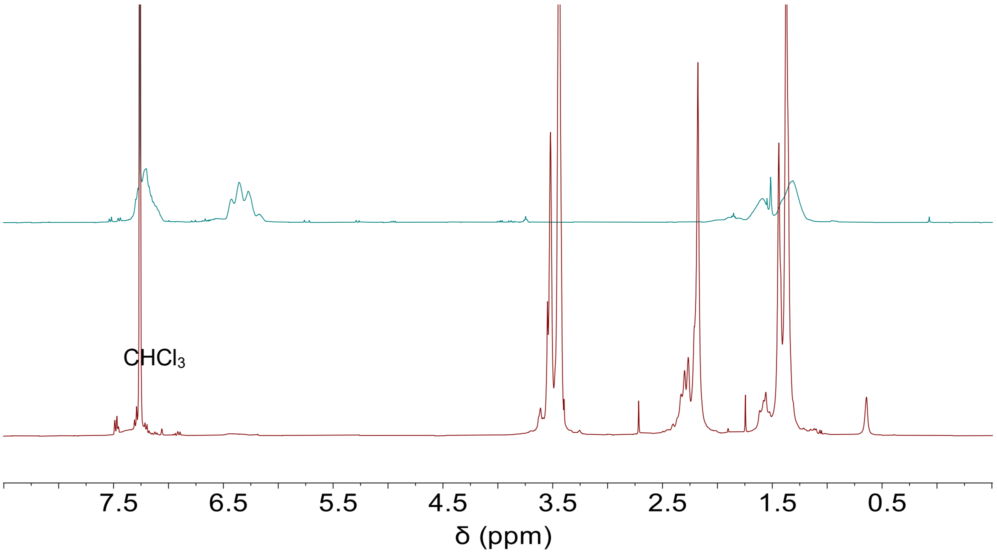

Figure S38. ^{1}H NMR spectra of PBS prepared via free radical polymerization (top) and the mixture obtained after precipitation following the grafting-from process with MMA after 24 hours (bottom, 400 MHz, $CDCl_3$). The persistence of peaks in the 6.0–6.5 ppm range, corresponding to aromatic bromides, indicates that PBS was not fully activated when using MMA as the monomer. This is likely attributed to the lower reducing potential of MMA (E_{red} = -2.28 V vs SCE) compared to PBS (E_{red} < -2.4 V vs SCE).[6]

References

[1] J. P. Cole, D.-F. Chen, M. Kudisch, R. M. Pearson, C.-H. Lim, G. M. Miyake, *J. Am. Chem. Soc.* **2020**, *142*, 13573-13581.
[2] V. K. Singh, C. Yu, S. Badgujar, Y. Kim, Y. Kwon, D. Kim, J. Lee, T. Akhter, G. Thangavel, L. S. Park, J. Lee, P. C. Nandajan, R. Wannemacher, B. Milián-Medina, L. Lüer, K. S. Kim, J. Gierschner, M. S. Kwon, *Nat. Catal.* **2018**, *1*, 794-804.
[3] Y. M. Lattke, D. A. Corbin, S. M. Sartor, B. G. McCarthy, G. M. Miyake, N. H. Damrauer, *J. Phys. Chem. A* **2021**, *125*, 3109-3121.
[4] T. Constantin, M. Zanini, A. Regni, N. S. Sheikh, F. Juliá, D. Leonori, *Science* **2020**, *367*, 1021-1026.
[5] a) J. Sobieski, A. Gorczyński, A. M. Jazani, G. Yilmaz, K. Matyjaszewski, *Angew. Chem. Int. Ed.* **2025**, *64*, e202415785; b) A. M. Jazani, G. Yilmaz, M. Baumer, J. Sobieski, S. Bernhard, K. Matyjaszewski, *J. Am. Chem. Soc.* **2025**, *147*, 12562-12573.
[6] F. Lorandi, M. Fantin, S. Shanmugam, Y. Wang, A. A. Isse, A. Gennaro, K. Matyjaszewski, *Macromolecules* **2019**, *52*, 1479-1488.
